# Salt responsive alternative splicing of a RING finger E3 ligase modulates the salt stress tolerance by fine-tuning the balance of COP9 signalosome subunit 5A

**DOI:** 10.1371/journal.pgen.1009898

**Published:** 2021-11-16

**Authors:** Yuan Zhou, Xiao-Hu Li, Qian-Huan Guo, Peng Liu, Ying Li, Chang-Ai Wu, Guo-Dong Yang, Jin-Guang Huang, Shi-Zhong Zhang, Cheng-Chao Zheng, Kang Yan

**Affiliations:** 1 State Key laboratory of Crop Biology, College of Life Sciences, Shandong Agricultural University, Tai’an, China; 2 Donald Danforth Plant Science Center, Saint Louis, Missouri, United States of America; Peking University, CHINA

## Abstract

Increasing evidence points to the tight relationship between alternative splicing (AS) and the salt stress response in plants. However, the mechanisms linking these two phenomena remain unclear. In this study, we have found that *Salt-Responsive Alternatively Spliced gene 1* (*SRAS1*), encoding a RING-Type E3 ligase, generates two splicing variants: *SRAS1*.*1* and *SRAS1*.*2*, which exhibit opposing responses to salt stress. The salt stress-responsive AS event resulted in greater accumulation of *SRAS1*.*1* and a lower level of *SRAS1*.*2*. Comprehensive phenotype analysis showed that overexpression of *SRAS1*.*1* made the plants more tolerant to salt stress, whereas overexpression of *SRAS1*.*2* made them more sensitive. In addition, we successfully identified the COP9 signalosome 5A (CSN5A) as the target of SRAS1. CSN5A is an essential player in the regulation of plant development and stress. The full-length SRAS1.1 promoted degradation of CSN5A by the 26S proteasome. By contrast, SRAS1.2 protected CSN5A by competing with SRAS1.1 on the same binding site. Thus, the salt stress-triggered AS controls the ratio of SRAS1.1/SRAS1.2 and switches on and off the degradation of CSN5A to balance the plant development and salt tolerance. Together, these results provide insights that salt-responsive AS acts as post-transcriptional regulation in mediating the function of E3 ligase.

## Author summary

High salinity severely affects plant growth and development, impairing crop production worldwide. E3 ligase is a stress-responsive regulator through ubiquitin-proteasome system for selective protein degradation. The E3s are regulated by transcriptional regulation and post-translational modifications. Here, we have discovered that stress-responsive AS acts as a post-transcriptional regulation modulating the function of E3 ligases. Intriguingly, the truncated proteins generated by salt-responsive AS play opposite roles compared with the full-length E3 ligase. The truncated isoform losing key domain could not degrade the target protein, instead, it interacts and competes with the E3 ligase through binding the same domain of the targets. This finding contributes significantly to a deeper mechanistic understanding of how AS regulates the function of E3 ligase in response to salt stress.

## Introduction

Environmental stress dramatically influences plant growth and development [1,2]. Salt stress is a major abiotic stress in agriculture around the world, resulting in growth inhibition, developmental changes, and reduction-in crop yield [3,4]. Stress signals are transduced to activate stress responsive genes and ion channel permeability [5]. To survive environmental stresses, plants evolved various strategies for perceiving external signals [1,6]. Several transcription factors are involved in plant responses to salt stress, including the CBF/DREB family, RD22BP, MYBs and ABF/AREB [7–9]. In addition, the SOS pathway can be activated by salt stress to maintain low Na^+^ and high K^+^ levels [4,10]. The phytohormone abscisic acid (ABA) plays a crucial role in plant responses aimed at coping with salt stress [6,11]. The expression of many genes that play multifaceted roles in salt stress response and tolerance is induced by elevated ABA levels when plants are stressed by high salinity [7]. Other salt-responsive genes encode proteins that function in damage limitation or repair, including oxidative stress-related genes, LEA/dehydrin-type genes, detoxification enzymes, chaperones and ubiquitination-related enzymes [6,12].

In eukaryotes, protein degradation by the ubiquitin (Ub)-mediated regulation pathway is a key mechanism involved modulating various cellular processes, including hormone signaling, DNA repair and biotic and abiotic stress responses [13,14]. E3 proteins, which are ubiquitin-protein ligases, catalyze attachment of Ub to their target proteins through sequential actions from E2 Ub-conjugating enzymes, and thus confer selectivity for a wide range of substrates [15]. A total of 1406 E3 genes have been identified in *Arabidopsis*, including 470 RING-type E3s [15–17]. RING-type E3-mediated protein degradation by the ubiquitin-26S proteasome system plays an essential role in response to salt stress [18–20]. Salt and Drought-Induced RING finger 1 (SDIR1) plays a key role in ABA signaling, ABA-related seed germination, and salt stress response [21]. The four members of the *Arabidopsis* ABA-insensitive ring protein (AIRP) family of RING-type E3 ubiquitin ligases, AIRP1, AIRP2, AIRP3 and AIRP4 are involved in regulation of ABA-mediated drought stress resistance [22,23].

Alternative splicing (AS), in which two or more mRNA variants are generated from a single pre-mRNA with multiple introns, is a common and fundamental process that expands the proteome and regulates mRNA levels in eukaryotes [24,25]. AS events were found in more than 60% of intron-containing genes in *Arabidopsis*, and this number is likely to increase under various developmental and environmental conditions as the number of high-throughput analyses and transcriptome data grows [24]. Individual mRNA variants generated from AS might play specific spatial or temporal roles in response to abiotic stress, thereby fine-tuning gene expression [26,27]. For example, *Heat Shock Factor A2* (*HsfA2*) can activate its own transcription by producing a truncated AS transcript, thus forming an autoregulatory loop [28,29]. Endoplasmic reticulum (ER) stress-triggered AS also plays roles in regulating *bZIP60*, which encodes a key transcription factor involved in the unfolded protein response (UPR) [30,31]. Remarkably, PP2C hypersensitive to ABA1 (*HAB1*) undergoes AS to produce two splice variants, encoding HAB1.1 and HAB1.2, which play opposing roles in ABA-mediated seed germination [32].

In recent years, a vast amount of data about AS have been produced by genome-wide studies in various organisms. However, despite the extensive AS data gathered, thus far, there is a gap between the large amounts of sequencing data and the functional identification of AS variants [24]. Consequently, the role of AS in regulating salt signaling and plant stress adaptations remains elusive. We are still in the early stage of understanding the potential mechanism of AS in response to environmental stresses [24].

In this study, we investigated the function of a RING-type E3 ligase gene, *SRAS1*, which generated two transcript isoforms that had opposing responses and divergent functions under salt stress. Our results provide novel insights into the AS-mediated post-transcriptional regulation of E3 ligase.

## Results

### Two isoforms of RING-type E3 ligase SRAS1 exhibit opposite responses to salt stress

**Fig 1:**
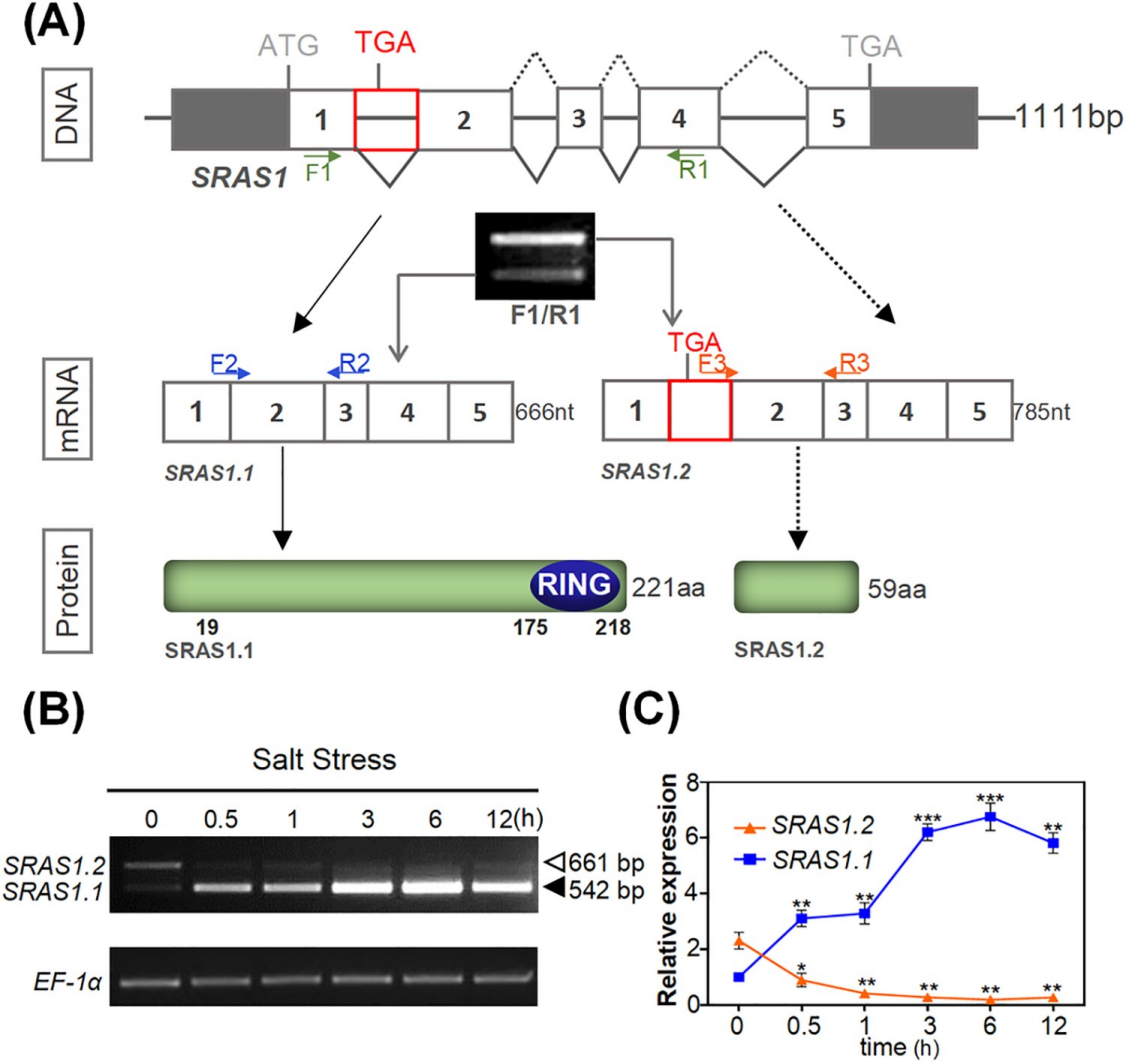
Alternative splicing of *SRAS1*. (A) Schematic diagrams of *SRAS1* gene. Two different intron splice sites are indicated in the gene diagram. Agarose gel electrophoresis band shows the AS events of *SRAS1*. Arrows (F1 and R1, F2 and R2, F3 and R3) indicate the location of primers used in RT-PCR and green color represents the exon region; blue region represents the RING domain. (B) RT-PCR analysis of the expression levels of *SRAS1*.*1* and *SRAS1*.*2* at 0, 0.5, 1, 3, 6 and 12 hours after 200 mM NaCl treatment. (C) qRT-PCR analysis of the expression of *SRAS1*.*1* and *SRAS1*.*2* at 0, 0.5, 1, 3, 6 and 12 hours after 200 mM NaCl treatment. Values are shown as the mean ± SE from three biological repeats. Statistically significant differences were identified between pairs of measurements using Student’s t-test (**P < 0.01, ***P < 0.001).

Recent studies have demonstrated that plant stress-related genes are particularly prone to AS events, which often modulate the ratio between active and non-active isoforms in response to abiotic stress [32,33]. To investigate the AS control of salt stress responses, we analyzed publicly available expression data through the Genevestigator mRNA-Seq platform (https://www.genevestigator.ethz.ch). This analysis identified the gene AT5g66070 as a salt-responsive gene (S1A Fig). AT5g66070 has been named *Arabidopsis* Tóxicos en Levadura 27 (*ATL27*), because it was originally assigned as a member of the E3 ubiquitin-protein ligase ATL subfamily [34]. And AT5g66070 has also been named ABA-related RING-type E3 ligase (*AtARRE*), as a negative regulator of ABA signaling [35]. In addition, the AT5g66070 transcripts are reported by TAIR (http://www.arabidopsis.org/) to undergo several AS events. Based on its salt inducibility, as well as on the capability of its transcript to undergo AS, we propose to rename this gene Salt-Responsive Alternatively Spliced gene 1 (*SRAS1*). *SRAS1* consists of 5 exons and 4 introns, and according to the TAIR database could generate five splice variants. In contrast to the documented information, by reverse transcription-PCR (RT-PCR) we only detected two distinctive transcript isoforms of *SRAS1* under salt stress. Intriguingly, the two AS transcripts exhibited opposite responses to salt stress (Fig 1A). Sequencing of the PCR products revealed that the bottom band was *SRAS1*.*1*, consisting of 666 nucleotides encoding the full-length SRAS protein with the RING domain, whereas the upper band was *SRAS1*.*2*, which encodes a truncated isoform generated from an intron retention (IR) splicing event involving the first intron (Fig 1A). *SRAS1*.*2* was a novel transcript and not documented in TAIR website. We identify SRAS1.2 as AT5G66070.6. This AS event introduced a premature termination codon (PTC) so that *SRAS1*.*2* encoded a small sized protein with 59 amino acids lacking the C-terminal RING finger domain (Fig 1A). To investigate the relationship between *SRAS1*.*1* transcript levels and salt stress in *Arabidopsis*, we performed RT-PCR analysis. Interestingly, levels of the *SRAS1*.*1* transcripts were elevated after 0.5 h of NaCl treatment and continued to increase with prolonged treatment, whereas the levels of *SRAS1*.*2* transcripts decreased dramatically as the duration of salt treatment increased (Fig 1B). Quantitative real-time PCR analyses were consistent with the above result, indicating that the *SRAS1* transcripts had opposite responses to salt stress (Fig 1C).

### The truncated isoform SRAS1.2 is not an active E3 ligase

**Fig 2:**
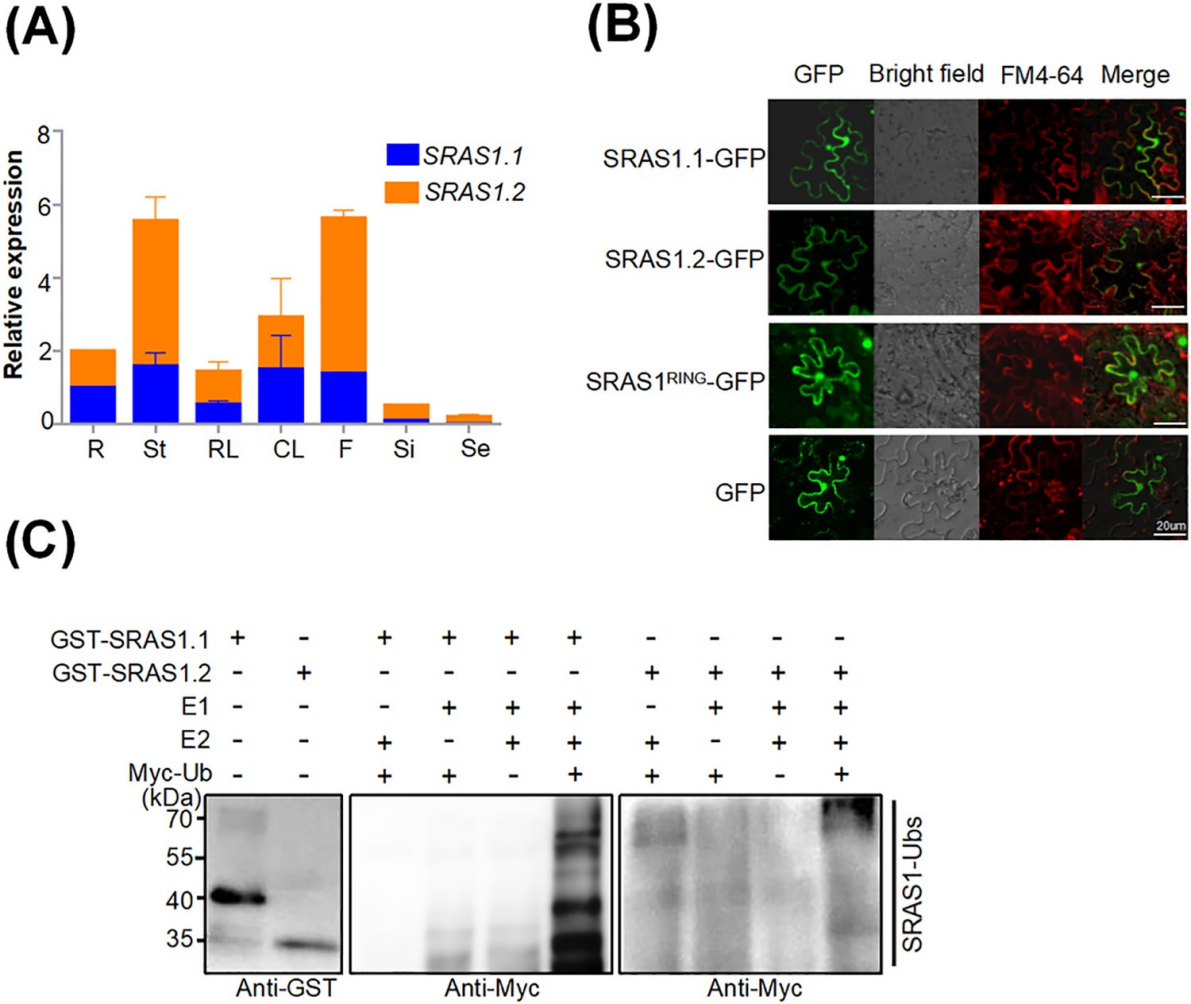
Subcellular localization and E3 ubiquitin ligase activity of SRAS1. qRT-PCR analysis of *SRAS1*.*1* and *SRAS1*.*2* expression levels in various tissues. Values are shown as the mean ± SE from three biological repeats. R: root, St: stem, RL: rosette leaf, CL: cauline leaf, F: flower, Si: silique, Se: seed. (B) Subcellular localization of SRAS1.1, SRAS1.2 and SRAS1.1^RING^. The SRAS1.1-GFP, SRAS1.2-GFP and SRAS1.1^RING^-GFP plasmids were transformed into tobacco leaf cells. The fluorescence signals were collected from tobacco epidermal cells. GFP fluorescence (left), FM4-64 staining (middle) and overlay images (right) are shown. Bars = 20 μm. (C) E3 ubiquitin ligase activity of SRAS1.1 and SRAS1.2. GST-SRAS1.1 and GST-SRAS1.2 proteins were assayed in the presence of El, E2 and Myc-Ubs. Ubiquitinated proteins were detected by immunoblotting with anti-GST and anti-Myc antibodies, respectively.

To detect spatial expression patterns of *SRAS1* transcripts, we performed quantitative real-time RT-PCR (qRT-PCR) analyses of RNA extracts from diverse plant tissues. The results revealed similar expression patterns of *SRAS1*.*1* and *SRAS1*.*2*. Expression levels were strongest in flowers and stems; moderate in cauline leaves, rosette leaves, and roots; and weakest in siliques and seeds (Fig 2A). These results confirm that *SRAS1* is constitutively expressed in various tissues; however, the expression levels of *SRAS1*.*2* are approximately 2-fold higher than *SRAS1*.*1* in flowers and stems, suggesting that the isoforms might have tissue-specific functions in different tissues.

Next, we examined the subcellular localization of the SRAS1.1 and SRAS1.2 proteins by transiently expressing SRAS1.1-GFP and SRAS1.2-GFP fusion proteins in *Nicotiana benthamiana* leaf cells. The fluorescence signals of SRAS1.1 and SRAS1.2 were detected in both cytoplasm and nucleus. The RING-finger domain was also localized in both the cytoplasm and nucleus (Fig 2B). These observations confirm that SRAS1.1 and SRAS1.2 have similar localizations (Fig 2B). Considering that SRAS1.2 is severely truncated and its GFP fusion protein might be diffusible, we also fused SRAS1.2 with a GFP dimer to increase its molecular weight and the subcellular localization remained the same (S1B Fig). SRAS1.1 consists of 221 amino acid residues, including a conserved RING domain in the C-terminus. RING-type proteins always function as E3 ubiquitin ligases [17]. By contrast, SRAS1.2, truncated at the C-terminus, carries no RING domain, leading us to investigate whether the SRAS1.2 still had E3 ligase activity or not. To answer this question, we fused SRAS1.1 and SRAS1.2 with the GST-tag to create GST-SRAS1.1 and GST-SRAS1.2, and subjected these proteins to an *in vitro* autoubiquitination assay (Fig 2C). In the presence of E1, E2 and Myc-ubiquitin, the GST-SRAS1.1 protein exhibited clear autoubiquitination, implying that SRAS1.1 exhibits E3 ligase activity *in vitro*. On the other hand, we detected no ubiquitination of GST-SRAS1.2 (Fig 2C). Taken together, these results indicate that SRAS1.1 is an active E3 ligase, but SRAS1.2 is not.

### SRAS1.1 is a regulator in salt stress signaling

**Fig 3:**
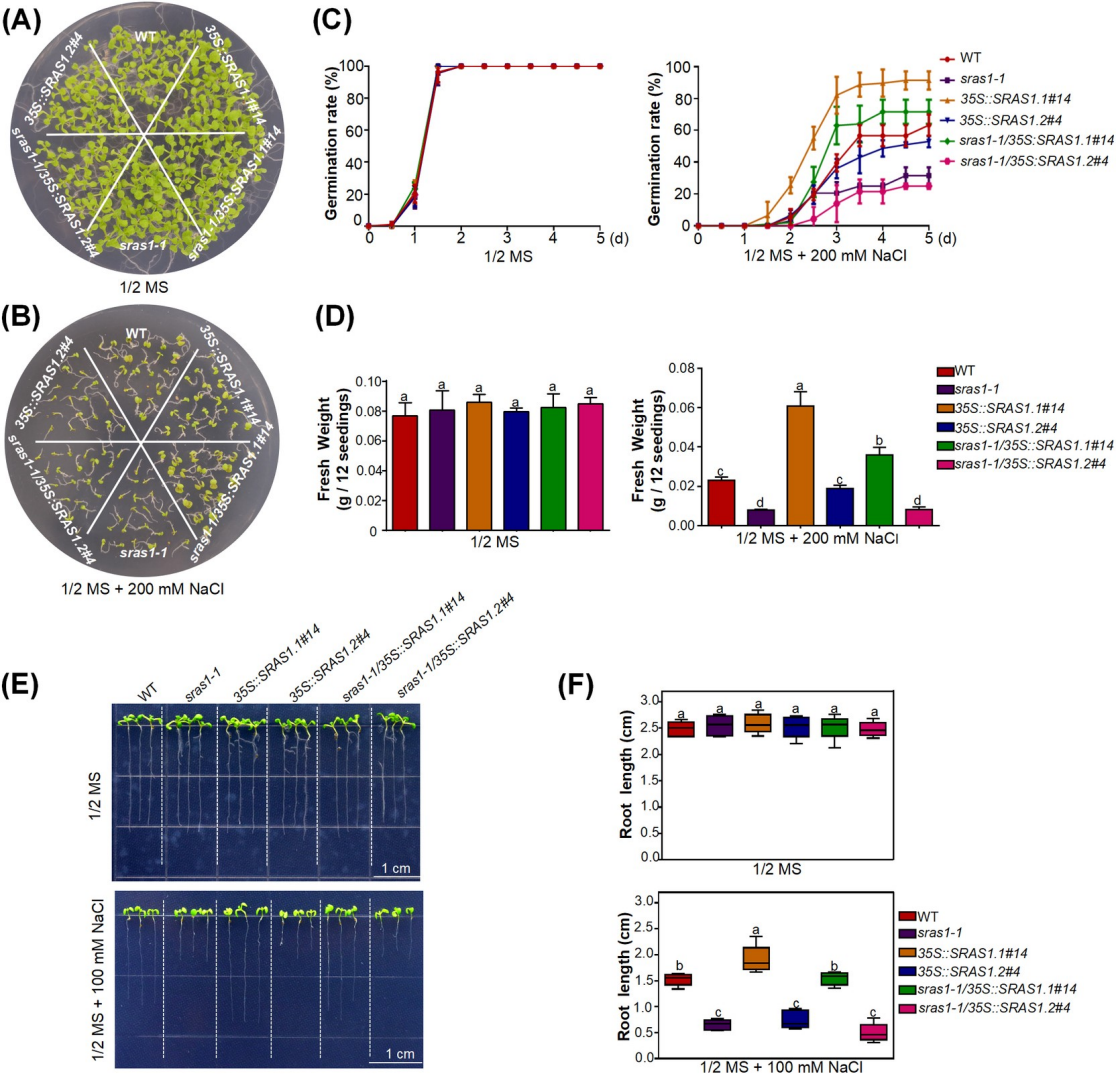
SRAS1 is involved in the salt stress response. Germination phenotype of WT, *sras1-1*, *35S*::*SRAS1*.*1*, *35S*::*SRAS1*.*2*, *sras1-1/35S*::*SRAS1*.*1* and *sras1-1/35S*::*SRAS1*.*2* seedlings grown on 1/2 MS medium. Images were taken 10 days after germination. Germination phenotype of WT, *sras1-1*, *35S*::*SRAS1*.*1*, *35S*::*SRAS1*.*2*, *sras1-1/35S*::*SRAS1*.*1* and *sras1-1/35S*::*SRAS1*.*2* seedlings grown on 1/2 MS medium with 200 mM NaCl. Images were taken 10 days after germination. Germination rates of WT, *sras1-1*, *35S*::*SRAS1*.*1*, *35S*::*SRAS1*.*2*, *sras1-1/35S*::*SRAS1*.*1* and *sras1-1/35S*::*SRAS1*.*2* seedlings grown on 1/2 MS medium with or without 200 mM NaCl. The values are the mean ± standard deviation from three biological replicates. (D) Fresh weight of WT, *sras1-1*, *35S*::*SRAS1*.*1*, *35S*::*SRAS1*.*2*, *sras1-1/35S*::*SRAS1*.*1* and *sras1-1/35S*::*SRAS1*.*2* seedlings. Error bars indicate SD (n = 60). Different letters indicate that values were significantly different at P < 0.01. (E) Root growth of WT, *sras1-1*, *35S*::*SRAS1*.*1*, *35S*::*SRAS1*.*2*, *sras1-1/35S*::*SRAS1*.*1* and *sras1-1/35S*::*SRAS1*.*2* in 1/2 MS medium. Seedlings grown in normal 1/2 MS medium for 3 d were transferred onto 1/2 MS medium with or without 100 mM NaCl and grown for another 7 d. Scale bar = 1 cm. (F) Measurement of root length of the plants shown in (E). Error bars = SE (n > 40 per genotype). Different letters indicate that values were significantly different at P < 0.01. The data shown are representative of three independent experiments.

To investigate the biological roles of *SRAS1* under salt stress, we ordered a *sras1-1* (Salk_034426) knockout mutant in which a T-DNA insertion in the 5’ untranslated region of the gene almost completely disrupted SRAS1.1 and SRAS1.2 expression (S2A Fig). Relative to wild-type (WT) plants, *sras1-1* mutants were more sensitive to salt stress (Fig 3A–3F). We also constructed overexpression transgenic lines in the Col-0 ecotype background. Two *35S*::*SRAS1*.*1* lines overexpressing full-length *SRAS1*.*1* CDS 666nt (*35S*::*SRAS1*.*1#14*, *35S*::*SRAS1*.*1#26*) and two *35S*::*SRAS1*.*2* lines overexpressing truncated *SRAS1*.*2* CDS 180nt (*35S*::*SRAS1*.*2#1*, *35S*::*SRAS1*.*2#4*) were used for further study (S2B and S2C Fig). WT, *sras1-1*, *35S*:*SRAS1*.*1* and *35S*:*SRAS1*.*2* overexpression transgenic seeds were germinated on half-strength Murashige and Skoog (1/2 MS) medium containing 0 or 200 mM NaCl. The germination rate of *SRAS1*.*1* overexpression lines was higher than that of WT, whereas *SRAS1*.*2* overexpression lines germinated less efficiently. In the presence of 200 mM NaCl, after 5 days, 28.5% of *sras1-1* seeds germinated, whereas 57.3% of WT, 86.9% of SRAS1.1 and 43.33% of SRAS1.2 seeds germinated (Figs 3A–3C and S2D and S2E). After the seedlings grew in 1/2 MS medium containing 200 mM NaCl for 14 days, the fresh weight of *sras1-1* and *SRAS1*.*2* overexpression lines was lower than that of WT, whereas the fresh weight of *SRAS1*.*1* overexpression lines was greater (Figs 3D and S2F and S2G). Seedlings grown in normal 1/2 MS medium for 3 d were transferred onto 1/2 MS medium with or without 100 mM NaCl and grown for another 7 d. Seedling root growth of *sras1-1* and *SRAS1*.*2* overexpression lines was reduced after salt treatment. And *sras1-1* mutants showed about 72% reduction in root growth relative to the untreated counterparts. Under the same salt conditions, *35S*::*SRAS1*.*1* lines grew better than the *sras1-1* mutant, the *35S*::*SRAS1*.*2* lines and the WT (Fig 3E and 3F). To achieve *SRAS1*.*2* overexpression with full-length cDNA, we also created point mutations at the 5’ splice site (from GU to AT) in the second intron to produce SRAS1.2, which forced retention of the second intron in the transcript (S2H Fig). The phenotypes of full-length *SRAS1*.*2* cDNA lines were similar to those of the lines overexpressing *SRAS1*.*2* CDS (S2I and S2G Fig).

To test whether SRAS1.1 and SRAS1.2 could rescue the mutant salt-sensitive phenotype, we generated *sras1-1/35S*::*SRAS1*.*1* and *sras1-1*/*35S*::*SRAS1*.*2* transgenic plants overexpressing SRAS1.1 and SRAS1.2 proteins in the *sras1-1* mutant background. When germinated on medium with salt stress, both *sras1-1* and the *sras1-1*/*35S*::*SRAS1*.*2* were more sensitive to salt stress than the WT in terms of both the germination rate, seedling fresh weight and root growth (Fig 3A–3F). In sharp contrast, *sras1-1*/*35S*::*SRAS1*.*1* plants were more tolerant to salt stress. In the presence of 200 mM NaCl, the fresh weight of *sras1-1* was reduced by ~64% relative to WT, whereas the fresh weight of *sras1-1*/*35S*::*SRAS1*.*2* plants was even lower than for *sras1-1*, indicating that the *sras1-1* mutant phenotype was only rescued by overexpressing *SRAS1*.*1*, not *SRAS1*.*2*. These results showed that overexpression of *SRAS1*.*1* made the plants more tolerant to salt stress, suggesting that SRAS1.1 plays a positive role in regulating plant resistance to salt stress.

### SRAS1 is involved in mediating salt-responsive gene expression

**Fig 4:**
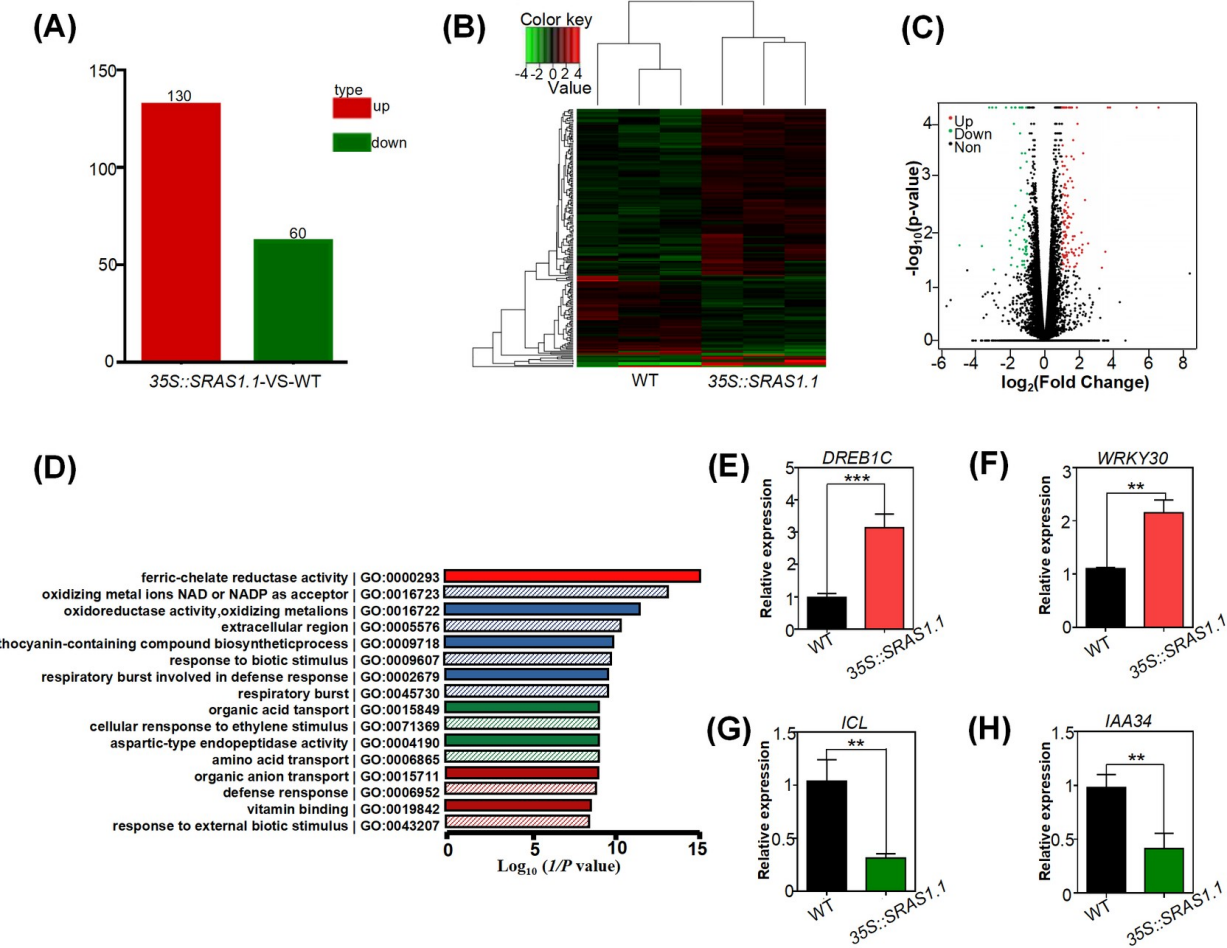
Genome-wide effects in *35S*::*SRAS1*.*1* transgenic plants. (A) Differentially expressed genes in RNA-seq assay. The histogram depicts the total number of differentially expressed genes in *35S*::*SRAS1*.*1* transgenic plants versus WT. Up- and down-regulated genes are shown in red and green bars, respectively. (B) Average linkage hierarchical clustering analysis of differentially expressed genes, with three repeated samples. (C) Scatter plots of genes in WT and *35S*::*SRAS1*.*1*. Red dots represent up-regulated genes, green dots represent down-regulated genes and black dots represent no significant difference genes. (D) GO function significant enrichment analysis. The log_10_(1/P) values represent the enrichment ratio of members of the specific GO terms. (E)-(G) qRT-PCR analysis of the expression levels of marker genes involved salt response pathways. Data are represented as means ± SD, n = 6. (**P < 0.01, ***P < 0.001).

To understand the transcriptional changes in the *SRAS1*.*1*-overexpressing lines, we conducted a transcriptome analysis using 14-day seedlings of the *35S*::*SRAS1*.*1* transgenic lines and the Col-0 controls. Three biological replicates were sequenced for each growth condition. The average read length aligned to the *Arabidopsis* reference genome (TAIR) was approximately 101 bp. Each library contained 23–24 million reads. Approximately 90% of reads mapped to unique loci, and 190 genes in *35S*::*SRAS1*.*1* transgenic plants were significantly differentially expressed relative to the WT (130 up-regulated and 60 down-regulated; criteria: P value < 0.001, fold change > 2; the details of each gene are provided in S1 Table) (Fig 4A–4C). We found that more genes were upregulated than downregulated. To evaluate the relationship between the genome-wide expression profiles, we performed linkage hierarchy clustering of differential expression between the WT sample and *SRAS1*.*1* samples.

Genomic analysis of the gene list showed that the genes induced by *SRAS1*.*1* were co-expressed with genes induced by ferric chelate reductase activity, oxidoreductase activity, and abiotic stress. Gene Ontology (http://www.geneontology.org/), Kyoto Encyclopedia of Genes and Genomes (http://www.genome.jp/kegg/) analyses revealed that upregulated genes were closely related to salt stress (GO0009607) and light stress (GO0016723). These genes can be divided into four categories according to their biological roles: molecular chaperones, energy factors, signal transmission, and metabolic factors (Figs 4D and S3A–S3C). We hypothesize that *SRAS1*.*1* regulates the expression of particular stress-resistance genes and light-related genes under high-salinity conditions. To validate the RNA-seq analysis, we checked the expression levels of differentially expressed genes by qRT-PCR (S3D and S3E Fig). Consistent with the RNA-seq data, positive regulators involved in salt response pathways, including *DRE/CRT-BINDING PROTEIN 1C* (*DREB1C*) and *WRKY DNA-BINDING PROTEIN 30* (*WRKY30*), were up-regulated in the *35S*::*SRAS1*.*1* transgenic plants relative to WT plants (Fig 4E and 4F). Meanwhile, some salt-responsive negative regulators, such as *ISOCITRATE LYASE* (*ICL*) and *INDOLE-3-ACETIC ACID INDUCIBLE 34* (*IAA34*), were down-regulated in the *35S*::*SRAS1*.*1* transgenic lines (Fig 4G and 4H). Taken together, these findings suggest that SRAS1.1 plays a role in salt stress signaling.

### SRAS1 physically interacts with CSN5A

**Fig 5:**
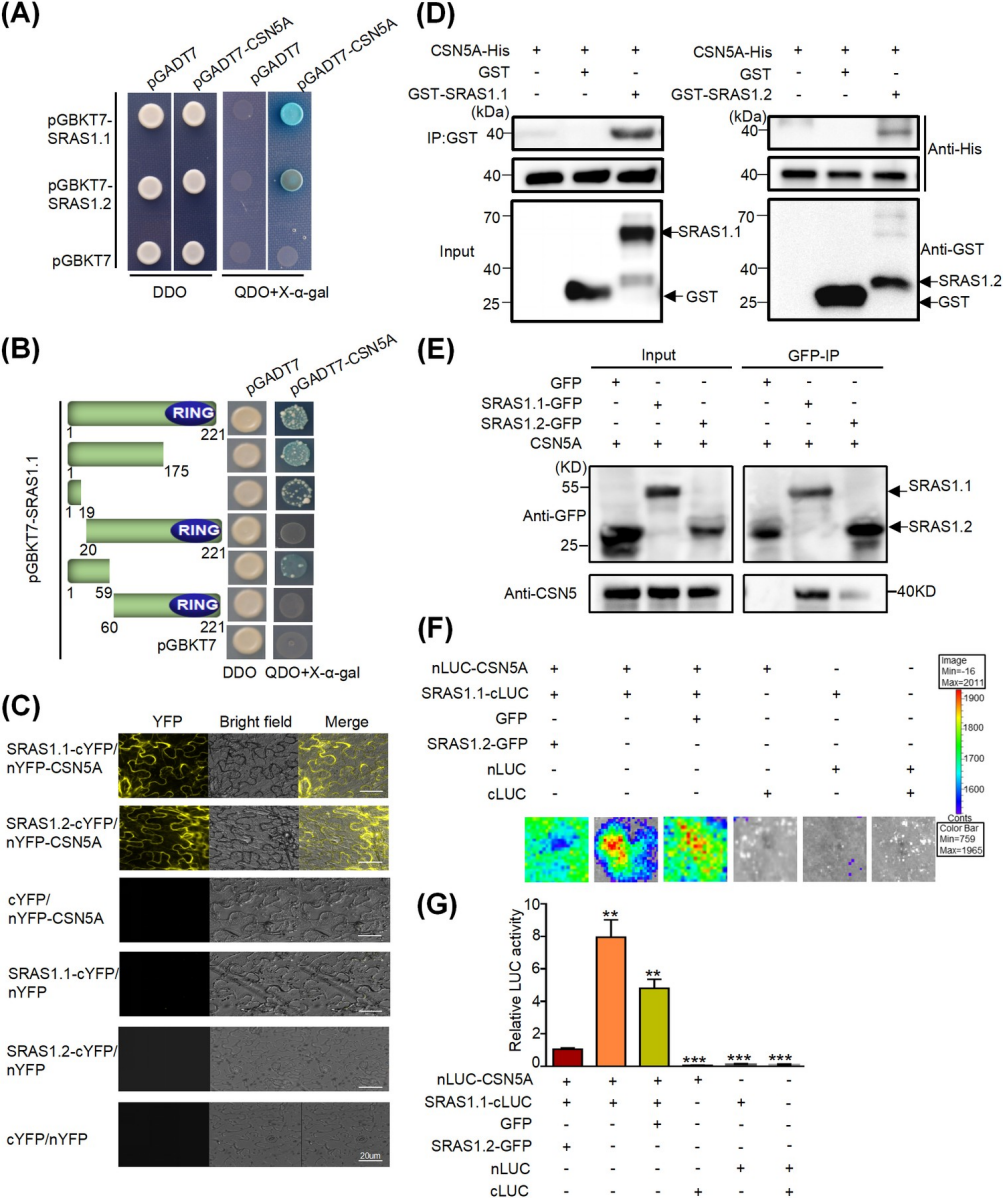
SRAS1 physically interacts with CSN5A. (A) Y2H assay demonstrating CSN5A interacts with SRAS1.1 and SRAS1.2. Yeast transformants were grown on the DDO media and on the QDO+X-α-gal, greenish blue indicates positive interactions. (B) Mapping the domain of SRAS1.1 that interacts with CSN5A. SRAS1.1 deletion constructs were made according to each domain. These deletion mutants were then tested for interaction in the Y2H system by co-transforming into Y2H Gold Yeast Strain. (C) BiFC assay of interaction of CSN5A with SRAS1.1 and SRAS1.2. Yellow fluorescence indicates positive interactions. cYFP and nYFP was used as a negative control, (Scale bar, 20 μm). (D) Pull-down assay of interaction of CSN5A with SRAS1.1 and SRAS1.2. Purified GST-SRAS1.1 and GST-SRAS1.2 proteins were immunoprecipitated with GST beads. Immunoprecipitated proteins were incubated with CSN5A-His and anti-His antibody was used to detect CSN5A-His. (E) Co-IP assay showing the interaction of CSN5A with SRAS1.1 and SRAS1.2 *in vivo*. The construct combinations were expressed in *N*. *benthamiana* leaves. Total proteins were extracted and immunoprecipitated with anti-GFP agarose beads. The proteins were detected with anti-GFP and anti-CSN5 antibodies. (F) LCI assay for analysis of the effect of SRAS1.2 on the interaction between SRAS1.1 and CSN5A in *N*. *benthamiana*. The color bar below shows the range of luminescence intensity in each image. The minus symbols (-) indicate empty vectors of nLUC, cLUC or GFP. (G) The quantification of LUC activity in *N*. *benthamiana* leaves. Data are represented as means ± SD, n = 6. **P < 0.01.

In general, RING-type E3 ligases function by ubiquitinating target proteins and triggering their degradation via the 26S proteasome [17,36,37]. To elucidate the regulatory mechanism of SRAS1 in response to salt stress, we performed a yeast two-hybrid (Y2H) screen to identify possible interacting proteins (S4 Fig). Based on this screen, we discovered that CSN5A was a putative interacting partner of SRAS1.1. CSN5A is an essential subunit of CSN5, which is an important unit of the COP9 signalosome (CSN), an evolutionarily conserved multiprotein complex that mediates light-regulated development and stress-linked resistance in plants and mammals [38,39]. There are two CSN5 subunits in *Arabidopsis*, CSN5A and CSN5B. The two subunits are assembled into distinct CSN complexes *in vivo*, which are present in different abundances; CSN5A appears to be predominant [40,41]. We found that the CSN family member CSN5A interacted with both SRAS1.1 and SRAS1.2 (Fig 5A). Due to the small size of the SRAS1.2 protein, we speculated that the binding of SRAS1.1 and SRAS1.2 to CSN5A was stimulated by the presence of the short N-terminal motif (Fig 5B). To test this hypothesis, we estimated the binding activity in a yeast two-hybrid system. The results revealed that amino acids 1–19 in the N-terminus of SRAS1 were critical for the interaction between SRAS1 and CSN5A (Fig 5B). To corroborate the interaction between SRAS1 and CSN5A, we performed a bimolecular fluorescence complementation (BiFC) assay. Reconstituted fluorescence was detected in the SRAS1.1-cYFP/nYFP-CSN5A and SRAS1.2-cYFP/nYFP-CSN5A samples, indicating that SRAS1.1 and SRAS1.2 interacted with CSN5A *in vivo* (Fig 5C). Next, we confirmed the physical interaction between SRAS1 and CSN5A by *in vitro* pull-down assay. CSN5A-His interacted with both GST-SRAS1.1 and GST-SRAS1.2 (Fig 5D). We also performed an *in vivo* coimmunoprecipitation (Co-IP) assay to check the interaction of CSN5A with SRAS1.1 and SRAS1.2 in plants. In these experiments, the 35S::SRAS1.1-GFP and 35S::SRAS1.2-GFP constructs were expressed in *N*. *benthamiana* through *Agrobacterium tumefaciens*-mediated infiltration. Total proteins were immunoprecipitated with an anti-GFP affinity gel matrix, and the bound proteins were eluted and subjected to immunoblot analysis with anti-GFP and anti-CSN5 antibodies. The results revealed that both SRAS1.1 and SRAS1.2 interacted with CSN5A (Fig 5E). In addition, an *in planta* luciferase complementation imaging (LCI) assay showed that co-expression of SRAS1.1 and SRAS1.2 with CSN5A generated strong luminescence signals that were not detected in the control pairs (Fig 5F and 5G). Collectively, these results indicate that SRAS1 physically interacts with CSN5A.

Some recent studies showed that AS-generated splicing isoforms from one gene could interact with each other in the form of dimers [28,32]. To test this possibility, we performed Y2H and BiFC assays, both of which confirmed that SRAS1.1 interacted with SRAS1.2 (S5A and S5B Fig).

### SRAS1.1 promotes CSN5A degradation by the 26S proteasome under salt stress

**Fig 6:**
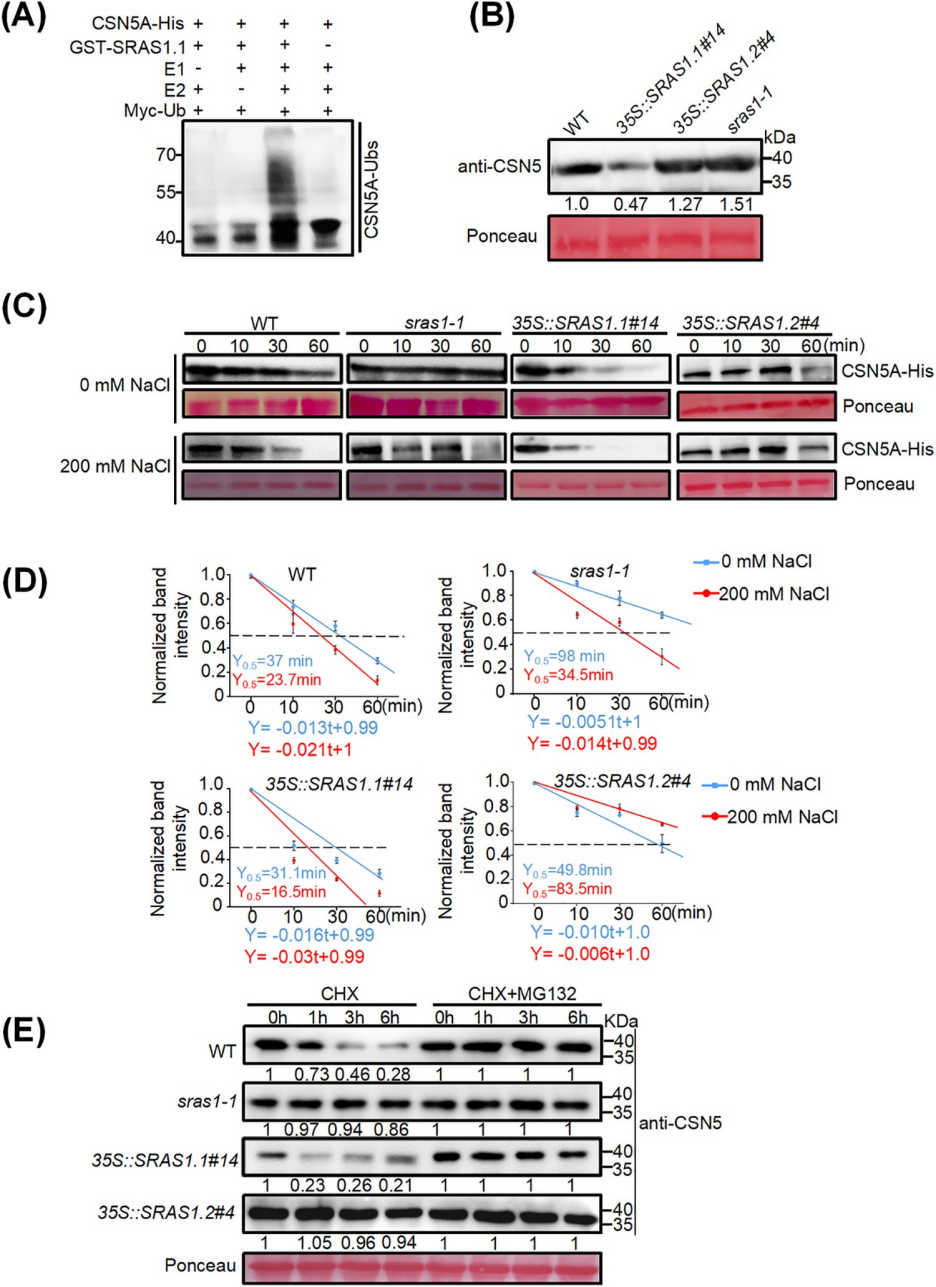
SRAS1.1 promotes degradation of CSN5A by the 26S proteasome. (A) *In vitro* ubiquitination assay shows CSN5A-His protein can be ubiquitinated by GST-SRAS1.1. Reaction products were analyzed by immuno-blotting with CSN5A-Ubs. (B) Immunoblot analysis of CSN5A protein levels in WT, *sras1-1*, *35S*::*SRAS1*.*1#14* and *35S*::*SRAS1*.*2#4*. Total protein was extracted from 7-day-old seedlings. Ponceau staining served as a loading control. (C) Cell-free assays showing the degradation rate of CSN5A-His incubated with the supernatant of WT, *sras1-1*, *35S*::*SRAS1*.*1#14* and *35S*::*SRAS1*.*2#4*. The degradation rate of CSN5A-His was detected by anti-His antibody. Ponceau staining of Rubisco indicates equal loading. (D) Normalized plot of CSN5A contents based on the band intensities shown in (C). Error bars indicate SEM (n = 3). (E) Immunoblot assay comparison of CSN5A degradation between the WT, *sras1-1*, *35S*::*SRAS1*.*1#14* and *35S*::*SRAS1*.*2#4*. The 7-day-old seedlings were treated with 100 μM CHX and 100 μM CHX+50 μM MG132 for different times. Total proteins were extracted and used for immunoblotting analysis with anti-CSN5 antibody.

To confirm our speculation that CSN5A is a substrate of SRAS1.1, we conducted an *in vitro* ubiquitination assay. CSN5A was expressed with a His tag in *E*. *coli*, and SRAS1.1 was expressed with a GST tag. The results revealed that CSN5A was ubiquitinated by SRAS1.1 in the presence of E1, E2 and ubiquitin. When any of these essential proteins was omitted from the reaction, the ubiquitinated form of CSN5A was not detected. These data directly demonstrate that CSN5A is a substrate of SRAS1.1 (Fig 6A).

Next, we performed western blotting to detect CSN5A protein levels in the WT, *SRAS1*.*1-*overexpressing, *SRAS1*.*2*-overexpressing and *sras1-1* lines. The results revealed that the levels of CSN5A protein were lower in the *SRAS1*.*1*-overexpressing lines than in the WT, *sras1-1* mutant and *SRAS1*.*2*-overexpressing lines (Fig 6B). Because *SRAS1*.*1* expression levels were very low under normal growth conditions, and this gene could be induced by NaCl treatment, we then evaluated the stability of CSN5A with or without NaCl treatment in the *SRAS1*.*1*-overexpressing, *sras1-1* mutant and *SRAS1*.*2*-overexpressing lines. A cell-free protein degradation assay showed that the levels of CSN5A protein were slightly lower in the WT in the absence of salt treatment, but decreased more rapidly in the *SRAS1*.*1*-overexpressing transgenic lines under the same condition, especially after 10 min (Fig 6C and 6D). By contrast, the CSN5A protein levels remained steady in the *sras1-1* mutant and *SRAS1*.*2*-overexpressing lines (Fig 6C). Under salt stress conditions, the degradation of CSN5A was significantly faster in *SRAS1*.*1*-overexpressing lines and the WT, but slower in the *SRAS1*.*2*-overexpressing lines (Fig 6C). CSN5A-His was much faster and had a shorter half-life in *35S*::*SRAS1*.*1#1*, with or without salt treatment (Fig 6C and 6D). However, there was no significant difference at the transcription level (S6A and S6B Fig). Taken together, these results demonstrated that the degradation of CSN5A by SRAS1.1 was accelerated under salt stress (Fig 6C and 6D). Hence, we hypothesized that the reduction in CSN5A levels in transgenic lines was due to degradation by 26S proteasome. To test this hypothesis, we investigated whether treating cells with the MG132 would attenuate the degradation of CSN5A. WT, *SRAS1*.*1*-overexpressing, *SRAS1*.*2*-overexpressing and *sras1-1* mutant lines were treated with the inhibitor, and total proteins were extracted. Nearly 78.5% of CSN5A disappeared after 1 hour. MG132 almost completely blocked degradation of CSN5A in the *SRAS1*.*1*-overexpressing transgenic lines, indicating that CSN5A was degraded by the 26S proteasome (Fig 6E). These data directly demonstrate that CSN5A degradation promoted by SRAS1.1 is dependent on the 26S proteasome pathway.

### SRAS1.2 suppresses the degradation of CSN5A by competing with SRAS1.1

**Fig 7:**
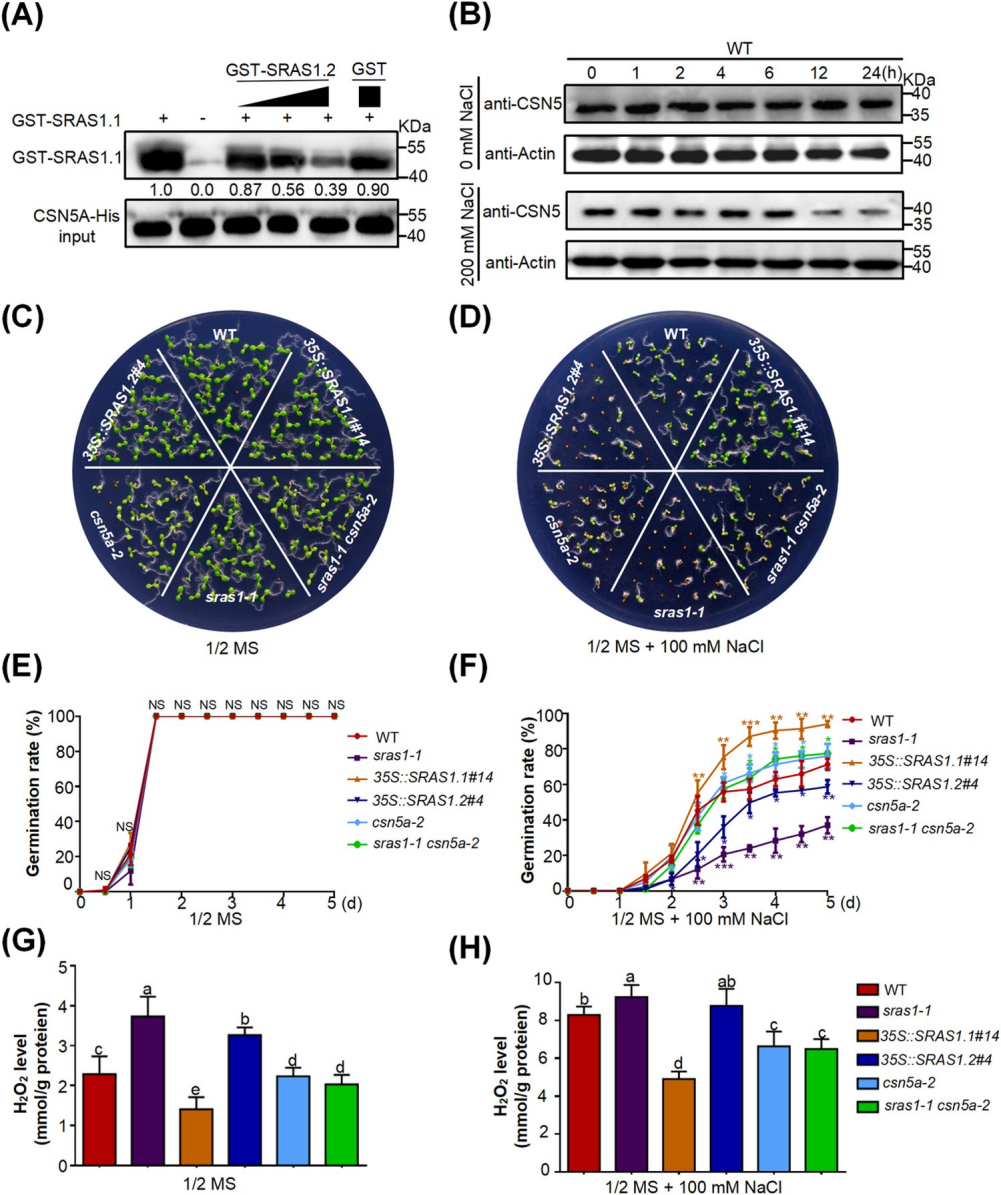
SRAS1.2 inhibits the degradation of CSN5A through affecting interactions between SRAS1.1 and CSN5A. *In vitro* pull-down assay displaying the changes in the interaction between SRAS1.1 and CSN5A by SRAS1.2 in a dose dependent manner. GST-SRAS1.1 protein combined with GST-SRAS1.2 or GST was incubated with immobilized CSN5A-His. The immunoprecipitated fractions were detected by anti-GST antibody. The gradient indicates an increasing amount of GST-SRAS1.2. CSN5A-His input is shown in the lower panel. (B) Immunoblot analysis of CSN5 protein levels under salt stress. Total protein extracted from 7-day-old WT seedlings treated with or without 200mM NaCl 0, 1, 2, 4, 6, 12 and 24 hours. The expression levels of CSN5 were detected with anti-CSN5 antibody. (C)-(D) Germination phenotype of WT, *sras1-1*, *35S*::*SRAS1*.*1*, *35S*::*SRAS1*.*2*, *csn5a-2* and *sras1-1 csn5a-2* seedlings grown on 1/2 MS medium with or without 100mM NaCl. Images were taken 7 days after germination. (E)-(F) Germination rates of WT, *sras1-1*, *35S*::*SRAS1*.*1*, *35S*::*SRAS1*.*2*, *csn5a-2* and *sras1-1 csn5a-2* seedlings grown on 1/2 MS medium with or without 100 mM NaCl. The values are the mean ± standard deviation from three biological replicates. (*P < 0.05, **P < 0.01, ***P < 0.001), NS, not significant (P ≥ 0.05). (G)-(H) H_2_O_2_ contents in WT, *sras1-1*, *35S*::*SRAS1*.*1*, *35S*::*SRAS1*.*2*, *csn5a-2* and *sras1-1 csn5a-2* seedings with or without 100 mM NaCl. Different letters indicate that values are significantly different at P < 0.01. The data shown are representative of three independent experiments.

The results described above raised an important question about the function of the truncated isoform SRAS1.2. The E3 ligase activity of SRAS1.1 is essential for its ability to promote CSN5A degradation under salt stress; however, SRAS1.2 also interacted with CSN5A even though it lacked E3 ligase activity. Therefore, we sought to determine what role SRAS1.2 plays in response to salt stress. We hypothesized that SRAS1.2 acted as a competitor that interferes with CSN5A degradation mediated by SRAS1.1. To test this idea, we performed a dose-dependent *in vitro* competitive pull-down experiment in which we investigated whether SRAS1.2 affected the interaction between SRAS1.1 and CSN5A. As shown in Fig 7A, increasing amounts of GST-SRAS1.2 decreased the amount of GST-SRAS1.1 bound to CSN5A-His, indicating that the interaction between CSN5A and SRAS1.1 was significantly weakened when SRAS1.2 was co-expressed with the other two proteins (Fig 7A). We performed western blotting to determine whether CSN5A protein levels are affected in response to salt stress (Fig 7B). Indeed, the levels of CNS5A gradually decreased during NaCl treatment (Figs 7B and S6C). Moreover, LCI assay showed that co-expression of SRAS1.2 and SRAS1.1 with CSN5A decrease the luminescence signal, implying SRAS1.2 affects the interaction between SRAS1.1 and CSN5A (Fig 5E and 5F). In Y2H assays, SRAS1.1 and SRAS1.2 share the same binding site of CSN5A (Fig 5B). For this reason, we propose that SRAS1.2 inhibits the degradation of CSN5A by competing with SRAS1.1.

### CSN5A acts downstream of SRAS1 to modulate salt stress response

CSN5A acts as a molecular switch between the stress response and development in *Arabidopsis* [42]. To validate the genetic hierarchy between *SRAS1* and *CSN5A*, we crossed the *sras1-1* and *csn5a-2* mutants and analyzed the phenotype under salt stress (Fig 7C–7F). In the presence of 100 mM NaCl, the germination rate of *csn5a-2* was higher than those of WT and *sras1-1* (Fig 7D and 7F). The germination percentage of *sras1-1 csn5a-2* seeds was comparable to that of *csn5a-2* seeds (Fig 7D and 7F). The *sras1-1* mutant is sensitive to salt stress, and *sras1-1 csn5a-2* double mutant rescues the *sras1-1* salt-sensitive phenotype (Fig 7D and 7F).

The transcriptome analysis showed that the expression levels of genes involved in oxidoreductase activity changed (Fig 4D). Thus, we detected the H_2_O_2_ level in WT, *SRAS1*.*1-*overexpressing, *SRAS1*.*2-*overexpressing, *sras1-1*, *csn5a-2* and *sras1-1 csn5a-2* lines under salt stress (Fig 7G and 7H). The cellular level of H_2_O_2_ was higher in *sras1-1* mutant seedlings than in WT seedlings, whereas the H_2_O_2_ level was lower in *csn5a-2* mutant seedlings. And the H_2_O_2_ level of *sras1-1 csn5a-2* seedlings was comparable to that of *csn5a-2*, indicating that CSN5A acted downstream of SRAS1 (Fig 7G and 7H).

**Fig 8:**
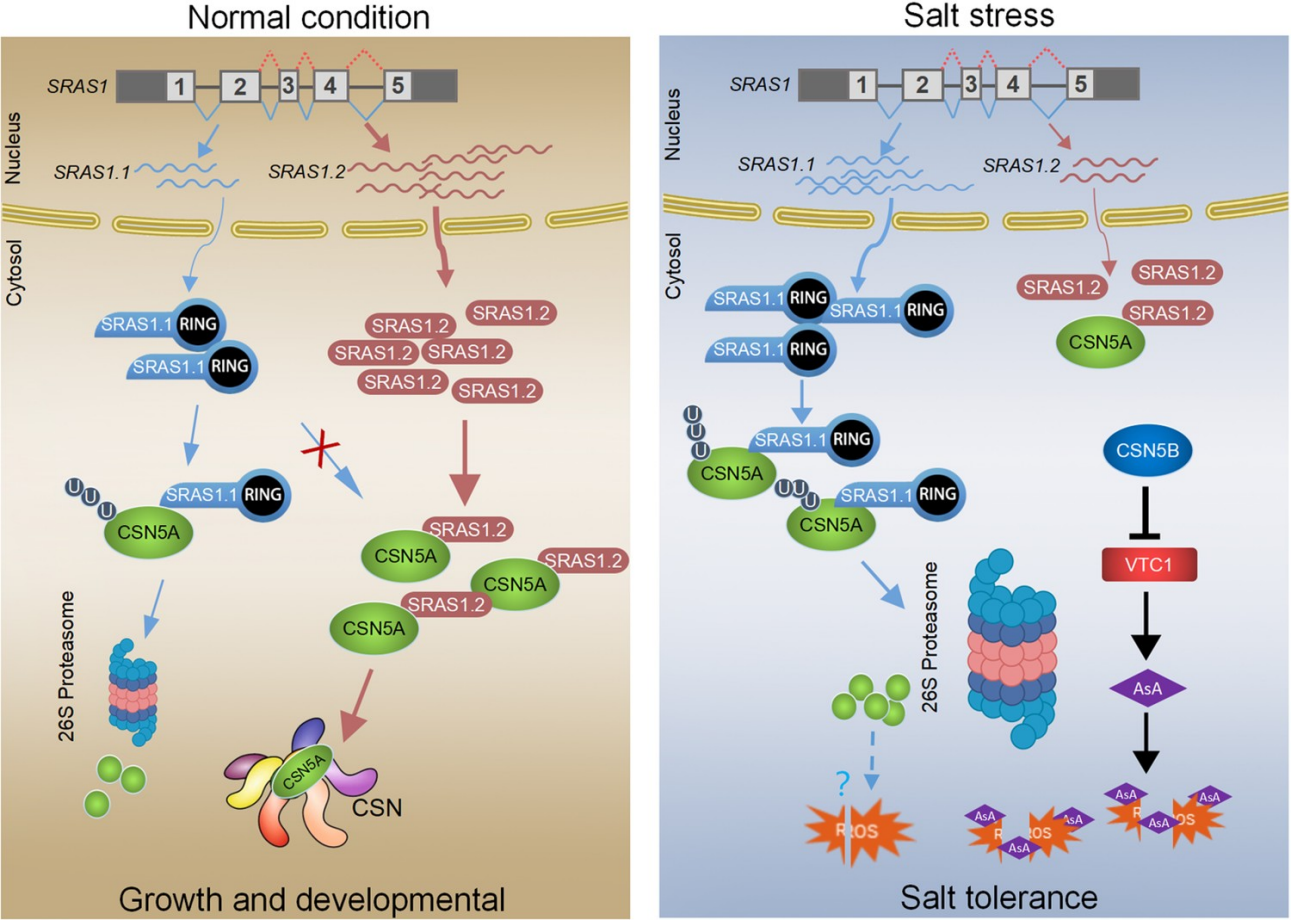
A regulatory model of salt responsive AS event on a E3 ligase SRAS1. The *SRAS1* could produce two splicing variants, encoding SRAS1.1 and SRAS1.2, respectively. The full-length SRAS1.1 protein has E3 ubiquitin ligase activity while the truncated SRAS1.2 lacks E3 ligase activity. Under normal conditions, *SRAS1* undergoes AS event to produce a high level of SRAS1.2 which interacts with and protects CSN5A. CSN5A is more stable, and incorporated into the CSN complex regulating plant growth and development. Salt stress that triggers splicing events affects the ratio of SRAS1.1/SRAS1.2, and SRAS1.1 promotes CSN5A degradation by 26S proteasome to control ROS accumulation. Simultaneously, CSN5B, which plays the predominant role, interacts with VTC1 in regulating ascorbic acid (AsA) synthesis to enhance salt tolerance.

Based on our findings, we propose a model illustrating the different roles of the AS-generated *SRAS1* transcripts to balance the CSN5A at the crossroads of growth and stress responses (Fig 8).

## Discussion

### Crucial roles of CSN5 in balancing plant development and salt stress tolerance

The CSN is the photomorphogenic complex regulating the activity of cullin-RING E3 ubiquitin ligases [43,44]. Among the eight CSN subunits, CSN5 subunit harbors the activity center of the CSN, binding numerous regulators and differentially affects the stability of each [41,45]. CSN5, encoded by CSN5A and CSN5B, is a key factor in plant development. The *Arabidopsis csn5a csn5b* double mutant has a lethal phenotype. The null mutant *csn5a* phenotype is less severe than the phenotype of *csn5a csn5b* double mutant, whereas the phenotype of *csn5b* mutant is similar with WT, suggesting that CSN5A is the predominantly functional subunit in plant development and growth [46–48]. The multiple functions of CSN5 have been extensively explored. The *csn5b* mutant is more tolerant to salt stress. CSN5B interacts with a GDP-mannose pyrophosphorylase VTC1 regulating ascorbic acid (AsA) synthesis in response to salt stress [46]. This investigation shows that the seeds of *csn5a* mutant are tolerant to salt stress than WT seeds, and the H_2_O_2_ level is lower in *csn5a* mutant seedlings (Fig 7C–7H). The detailed mechanism on how CSN5A controls ROS accumulation is still not clear. However, we speculate that this may be related to the role of CSN5A in ABA signaling. The previous study showed that CSN5A regulated seed germination by facilitating degradation of the RGL2 and ABI5 proteins [42]. Together, these observations revealed that CSN5A tightly modulates plant growth and salt stress responses. It should be noted that the *csn5b* mutant is more tolerant to salt stress than *csn5a*, implying that CSN5B plays the predominantly role in salt resistance.

This raises the question of how CSN5A is regulated between plant growth and stress resistance. In this study, we have found that the salt-responsive AS of the E3 ligase SRAS1.1 fine-tunes the CSN5A degradation. The salt stress mediates the ratio of SRAS1.1/SRAS1.2 to switch on and off the degradation of CSN5A (Fig 8).

To test whether SRAS1.1 interacts with CSN5B, Y2H and Co-IP assays were performed. We found that SRAS1.1 interacted with CSN5B in yeast, but SRAS1.1 did not interact with CSN5B in plants (S7A and S7B Fig). We speculated that CSN5A and CSN5B share high sequence similarity. A cell-free protein degradation assay showed that the CSN5B protein levels remained steady in *SRAS1*.*1*-overexpressing lines, indicating that SRAS1.1 did not affect the CSN5B degradation (S7C and S7D Fig).

### Emerging roles of truncated splicing isoforms in environmental stress adaptation

The ubiquitin-proteasome system (UPS), including ubiquitin (Ub), E1, E2, E3, 26S proteasome, is a rapid mechanism for selective protein degradation and plays crucial roles in plant development and stress tolerance [14,15]. The E3 ligase genes are stress-responsive, resulting in the altered ubiquitination and degradation of target proteins [36,49]. With more than 500 predicted members, the RING E3s constitute the largest ligases family [33]. The RING E3s are controlled by transcriptional regulation and post-translational modifications [17]. Here, we demonstrated that stress-responsive AS acted as a post-transcriptional regulation modulating the function of E3 ligases in two ways. First, the truncated isoform losing RING domain could not degrade the target protein, instead, it played the opposite role by interacting and protecting the target protein. We propose that the truncated isoform competes with the full length E3 through the same binding site. Second, AS changed the ratio of two transcripts on demand in response to environmental stresses. Immunoblotting analysis showed that the protein levels of SRAS1.1 and SRAS1.2 remained steady under salt stress (S8A and S8B Fig). We propose that post-transcriptional regulation is a major factor affecting the expression of SRAS1.1 and SRAS1.2.

Notably, the recent studies showed that several truncated isoforms generated by AS lost key domains and exhibited diverse and powerful functions under stresses [50–52]. For example, a truncated splice form produced by heat-induced AS activates the *HsfA2* promoter and positively autoregulates its own transcription [28]. HAB1.2, a truncated HAB1 PP2C isoform that lacks all phosphatase activity, is unable to transduce the stress signal via the ABA pathway [32]. However, little is known about functional splicing variants produced in response to salt stress. To determine how many salt-responsive AS genes exist, we analyzed and found the transcripts of 237 reported genes strongly responded to salt stress in *Arabidopsis*. About 28% of them (67 genes) had splicing variants annotated in the TAIR database. 41 *SRAS* genes were identified including *SRAS1* in this study (S2 Table). The functions of AS-triggered truncated proteins will be investigated in our future work.

### How salt stress triggered and affected AS

The recent studies showed that AS was a significant regulatory mechanism in response to salt stress [24,26,53,54]. However, exactly how salt stress controls AS isoform ratios and the timing of the mechanism in response to environmental signals remains elusive. In mammals, increasing evidence points to epigenetic and epitranscriptome changes, such as chromatin structure, DNA methylation, histone modifications and transcription elongation rate [55,56]. In plants, the role of epigenetic modifications in regulating transcription rate and mRNA abundance under stress is beginning to emerge [24,56]. Ullah *et al*. indicated that the chromatin structure was more open in retained introns, suggesting that the open chromatin architecture enhanced the pol II elongation rate, which led to skipping of splice sites [57]. Floral initiator Shk1 kinase binding protein1 (SKB1) associated with chromatin and thereby increases H4R3sme2 (histone 4 arginine 3 symmetric demethylation) levels to confers high salt tolerance by regulating transcription and pre-mRNA splicing [58]. On the other hand, emerging evidence also indicates that splicing factors play key roles in stress-responsive pre-mRNA splicing and AS, unveiling a novel regulatory layer in plant stress tolerance [59]. Genome-wide analysis and genetic results revealed that SKIP, a novel splicing factor, was required for the AS and mRNA maturation of lots of salt tolerance genes [60,61]. SR45a, a member of the conserved SR family, directly interacted with the cap-binding complex to promote salt stress responsive gene regulation [62]. Elucidation of the precise manner in salt-triggered AS will be investigated in the future work.

In this study, an example is provided that salt responsive AS could be a regulatory mechanism modulating the levels of E3 ligases. It would be interesting to investigate how widespread this mechanism is, and the molecular dissection of the AS-generated *SRASs*. Such studies will provide insights and details into the stress-responsive AS regulation.

## Materials and methods

### Plant material and growth conditions

*Arabidopsis* (*Arabidopsis thaliana*) plants used in this study were in the Columbia-0 background. The T-DNA insertion lines *sras1-1* (SALK_034426) and *csn5a-2* (SALK_027705), were obtained from the *Arabidopsis* Biological Resource Center (https://abrc.osu.edu/). All the mutants were confirmed by RT-PCR (primers were listed in S3 Table). Seeds were sterilized and then plated on 1/2 MS medium (pH 5.8; Sigma-Aldrich, St. Louis, MO, USA) containing 1.5% agar. After stratification in the dark at 4°C for 3 days, the plates were transferred to a growth chamber with long-day conditions (LDs; 16-h light/8-h dark cycles) at 22°C. After 7 days, the seedlings were potted in soil and placed in a growth chamber.

### Salt stress treatment

For the germination assays, 30 seeds for each line were sterilized and plated on 1/2 MS medium or 1/2 MS medium containing 200 mM or 100mM NaCl after 3 days of stratification. For each germination assay, biological triplicates were performed. For the fresh weight assay, seedlings were grown on 1/2 MS medium or 1/2 MS medium containing 200 mM NaCl after 14 days, seedlings were collected and weighed. For the root growth, seedlings grown in normal 1/2 MS medium for 3 d were transferred onto 1/2 MS medium with or without 100 mM NaCl and grown for another 7 days.

### Plasmid construction and plant transformation

The CDS of *SRAS1*.*1* and *SRAS1*.*2* were amplified by PCR from WT cDNA, then subcloned into the plasmid pBI121 (S4 Table). After verification by sequencing, the binary vector was introduced into *Agrobacterium tumefaciens* strain GV3101. The strains carrying different constructs were used to transform WT or *sras1-1* plants using the floral dip method. For selection of transgenic plants, the T0 seeds were sterilized and germinated on agar medium containing 50 mg/L kanamycin or 25 mg/L hygromycin. T3 homozygous lines were used for further study.

### RNA extraction and RT-PCR, qRT-PCR

Total RNA was extracted from 7-day-old seedlings of WT and transgenic plants before and after treatment with 200 mM NaCl or specific organs of WT using the TRIzol reagent (Invitrogen, Carlsbad, CA, USA). The RT-PCR and qRT-PCR assays were performed as described previously [63]. The *UBQ10* reference gene was used as an internal control. Intensity of gel bands were quantified using ImageJ software (http://imagej.nih.gov/ij/).

### RNA-seq analysis

Total RNA was isolated with TRIzol (Invitrogen) from 2 week old seedlings of the WT and *35S*::*SRAS1*.*1* overexpression lines grown on 1/2 MS medium. The transcriptome analysis was performed by CapitaBio Technology with three biological replicates. Library construction was performed according to illumina standard instructions. Reads were aligned to the *Arabidopsis* genome using TopHat2. Genes with adjusted P < 0.01 were considered to be differentially expressed. We uploaded the transcriptome data in the National Center for Biotechnology Information Sequence Read Archive (PRJ NA735616).

### Subcellular localization

For transient expression in *N*. *benthamiana* leaf cells, the *SRAS1*.*1* and *SRAS1*.*2* coding regions and an individual RING domain (175–218 aa) were fused in the frame to the coding region for the C-terminal GFP driven by the CaMV35S promoter. For the larger GFP fusion protein, we constructed plasmids 2×GFP which express 2 copies in tandem of GFP, under the cytomegalovirus (CMV) promoter, then *SRAS1*.*2* coding regions were fused in the frame. The GFP dimer vector was constructed using the method described in references [64]. The constructs were infiltrated into *N*. *benthamiana* plant leaves. After 3 days, fluorescence was observed with a Zeiss LSM880 confocal microscope (Zeiss, Germany) at 488 nm. The amphiphilic styryl dye FM4-64 was used as a PM marker.

### LCI assays

The full-length CDS of *SRAS1*.*1* was cloned into the pCAMBIA1300-cLUC (cLUC) vector to generate the SRAS1.1-cLUC construct, the full-length CDS of *CSN5A* was cloned into the pCAMBIA1300-nLUC (nLUC) vector to generate the nLUC-CSN5A construct, and the full-length CDS of *SRAS1*.*2* was cloned into the pROKII-GFP vector to generate the SRAS1.2-GFP construct. Next, 1 ml samples of *Agrobacterium tumefaciens* cells harboring nLUC-CSN5A, SRAS1.1-cLUC, or SRAS1.2-GFP were mixed equally to obtain the following combinations, each with a final optical density at 600 nm (OD600 = 1.5): nLUC-CSN5A/cLUC, nLUC-CSN5A/SRAS1.1-cLUC, nLUC-CSN5A/SRAS1.1-cLUC/GFP, nLUC-CSN5A/SRAS1.1-cLUC/SRAS1.2-GFP, nLUC/SRAS1.1-cLUC and nLUC/cLUC. Each combination of *A*. *tumefaciens* cells was infiltrated separately into *N*. *benthamiana* leaves and expressed for 48 h. The signals were detected by CCD (Olympus BX51).

### BiFC assays

The full-length coding sequences of *SRAS1*.*1*, *SRAS1*.*2*, *CSN5A* genes were amplified and cloned into BiFC vector pSET-n/cYFP. The SRAS1.1-cYFP/nYFP-CSN5A, SRAS1.2-cYFP/nYFP-CSN5A, cYFP/nYFP-CSN5A, SRAS1.1-cYFP/nYFP, SRAS1.2-cYFP/nYFP and cYFP/nYFP constructs were transformed into *N*. *benthamiana* leaves and expressed for 72 h, and then the fluorescence was detected by confocal microscopy (Zeiss, Germany).

### Y2H assays

To examine SRAS1.1 and SRAS1.2 interaction with CSN5A and CSN5B, the expression constructs were co-transformed into yeast Y2H Gold Yeast Strain cells and transformed cells were selected by growth on SD-Leu-Trp (DDO) medium and SD-L-W-H-Ade (QDO).

### Protein pull-down assays

*In vitro* pull-down and competitive pull-down assays, CSN5A-His (50 μg) and GST-SRAS1.1 (50 μg) or CSN5A-His (50 μg) and GST-SRAS1.2 (50 μg) were mixed together and incubated for 2 h at 4°C with constant rocking in 1 mL of binding buffer (50 mM Tris-HCl, 150 mM NaCl, pH 8.0). Afterwards, GST proteins were purified with a Pierce Glutathione Spin Column, eluted and analyzed with anti-His antibody (CWBIO, Beijing, China), following the protocol described previously [65].

### Co-IP assays

For Co-IP experiments, the plasmid pairs 35S::SRAS1.1-GFP/35S::CSN5A, 35S::SRAS1.2-GFP/35S::CSN5A and 35S:: GFP/35S::CSN5A were co-infiltrated into the *N*. *benthamiana* leaves. At approximately 48 h, the leaves were collected and homogenized in protein extraction buffer (50 mM Tris-MES, pH 8.0, 0.5 M sucrose, 1 mM MgCl_2_, 10 mM EDTA, 5 mM DTT, 1 mM phenylmethylsulfonyl fluoride). After protein extraction, anti-GFP antibodies (Transgene; Beijing, China 1:300 dilution) coupled to magnetic beads were mixed with protein samples and incubated at 4°C for 5–6 h. The captured proteins were separated by SDS-PAGE. Anti-GFP (1:1,000 dilution), anti-CSN5 (abcam; Shanghai, China 1:3,000 dilution) were used to detect SRAS1.1, SRAS1.2 and CSN5 respectively.

### Cell-free degradation assays

To investigate the effects of SRAS1.1 on CSN5A, we determined CSN5A-His protein levels after incubation with total protein extracts from WT, *35S*::*SRAS1*.*1#14*, *35S*::*SRAS1*.*2#4* and *sras1-1* seedlings grown on 1/2 MS medium, following the protocol described previously [63]. CSN5A-His protein was purified from *E*. *coli*.

### *In vitro* ubiquitination assays

The *in vitro* ubiquitination assays were performed as described previously [66]. In brief, A 100 ng quantity of wheat (Triticum aestivum) E1, 200 ng of purified E2, 5 mg of Myc-tagged ubiquitin (Boston Biochemicals, Cambridge, MA, USA), 1 mg of purified GST-SRAS1.1, GST-SRAS1.2 and CSN5A-His or CSN5B-His were added to 30 μl of ubiquitination reaction buffer (50 mM Tris-HCl pH 7.5, 2 mM ATP, 5 mM MgCl_2_, 2 mM DTT). After 24 h at 30°C the reactions were stopped by adding 5×loading buffer, the samples were then boiled at 100°C for 5 min. The products were electrophoresed on a 15% SDS polyacrylamide gel electrophoresis (PAGE) gel and detected with anti-His (CWBIO, Beijing, China) and anti-Myc (CWBIO, Beijing, China) antibodies by western blotting.

### Quantitation of hydrogen peroxide radical

To measure the content of H_2_O_2_ in WT, *sras1-1*, *35S*::*SRAS1*.*1*, *35S*::*SRAS1*.*2*, *csn5a-2* and *sras1-1 csn5a-2* seedlings with or without 100 mM NaCl, the corresponding assay kits (Keming, Suzhou, China) were used to measure the content of H_2_O_2_. The H_2_O_2_ content was measured using a previously reported method [67].

## Supporting information

S1 Fig *SRAS1* responses to salt stress. (A) Expression analysis of *SRAS1* genes in various tissues under salt stress in *Arabidopsis*. Expression analysis was carried out with mRNA-seq datasets using Genevestigator. R: root, St: stem, RL: rosette leaf, CL: cauline leaf, F: flower, Si: silique, Se:seed. (B) Subcellular localization of SRAS1.2. The SRAS1.2-GFP, SRAS1.2–2×GFP plasmids were transformed into tobacco leaf cells. The fluorescence signals were collected from tobacco epidermal cells. GFP fluorescence (left) is shown. Bars = 20μm.
(TIF)

S2 Fig Phenotypes of *35S::SRAS1.1* and *35S::SRAS1.2* transgenic plants under salt stress. (A) RT-PCR analysis of the *SRAS1*.*1* and *SRAS1*.*2* transcripts in WT, *sras1-1* mutant plants. *EF-1α* was used as a loading control. (B) qRT-PCR analysis of the expression of *SRAS1*.*1* in WT, *35S*::*SRAS1*.*1#14* and *35S*::*SRAS1*.*1#26* seedlings. *UBQ10* was used as a reference gene. Values are mean ± SE from three biological repeats. (C) qRT-PCR analysis of the expression of *SRAS1*.*2* in WT, *35S*::*SRAS1*.*2#1* and *35S*::*SRAS1*.*2#4* seedlings. *UBQ10* was used as a reference gene. Values are mean ± SE from three biological repeats. (D) Germination phenotype of WT, *35S*::*SRAS1*.*1#14*, *35S*::*SRAS1*.*1#26*, *35S*::*SRAS1*.*2#1*, *35S*::*SRAS1*.*2#4* and *sras1-1* seedlings grown on 1/2 MS medium with or without 200 mM NaCl. Images were taken 7 days after germination. (E) The germination rates of WT, *35S*::*SRAS1*.*1#14*, *35S*::*SRAS1*.*1#26*, *35S*::*SRAS1*.*2#1*, *35S*::*SRAS1*.*2#4* and *sras1-1* seedlings grown on 1/2 MS medium with or without 200 mM NaCl. The values are the mean ± standard deviation from three biological replicates. (F) Fresh weight of *35S*::*SRAS1*.*1*, *35S*::*SRAS1*.*2*, WT and *sras1-1* seedlings growing 1/2 MS agar plates with or without 200 mM NaCl photographs were taken after growing at 22°C for 14 d. Bar = 1 cm. (G) The quantification of relative fresh weight of *35S*::*SRAS1*.*1*, *35S*::*SRAS1*.*2*, WT and *sras1-1* seedlings growing 1/2 MS agar plates with or without 200 mM NaCl. The bars indicate means ± SD of three independent measurements. Different letters indicate that values were significantly different at P < 0.01. (H) Schematic illustration of the T-DNA insertion sites in the *sras1-1* mutants. Gray boxes represent untranslated regions (UTRs), White boxes represent exons; line segments represent introns; black arrows represent insertion sites of the *sras1-1* mutant alleles. GU or AT represent special mutation points in overexpressing *SRAS1*.*2* transcript. (I) Germination phenotype of WT, *sras1-1 35S*::*SRAS1*.*2#4* and *35S*::*SRAS1*.*2 cDNA(AT)* seedlings grown on 1/2 MS medium with or without 200 mM NaCl. Images were taken 7 days after germination. (J) Fresh weight of WT, *sras1-1*, *35S*::*SRAS1*.*2#4* and *35S*::*SRAS1*.*2 cDNA(AT)* seedlings. Error bars indicate SD (n = 60). Different letters indicate that values were significantly different at P < 0.01.
(TIF)

S3 Fig Genome-wide effects in *35S::SRAS1.1* transgenic plants. (A) Enrichment of differential genes in KEGG classification. (B) GO function significant enriched pathway terms. The abscissa is the negative log value of p-value, the ordinate is the first 30 enriched GO terms and function descriptions. (C) Gene ontology second-level entry frequency chart. The abscissa is the GO database function description, the ordinate is the number of genes. Different biological functions are shown in red, blue and green bars, respectively. (D)-(E) qRT-PCR analysis of the expression levels of marker genes involved salt response pathways. Data are represented as means ± SD, n = 6. **P < 0.01**P < 0.001.
(TIF)

S4 Fig A Y2H screen to identify SRAS1.1 interacting proteins. Interactions between SRAS1.1 and target proteins in Y2H assays. Yeast transformants were grown on the DDO media and on the QDO+X-α-gal, greenish blue indicates positive interactions, greenish blue indicates positive interactions. (B) Detailed information of SRAS1.1 interaction proteins. The SRAS1.1-interacting proteins, plasmids were recovered from yeast strains showing positive interactions, and their sequences were verified by DNA sequencing. Sequence data for the proteins described found in the *Arabidopsis* TAIR database.
(TIF)

S5 Fig SRAS1.1 physically interacts with SRAS1.2. (A) Y2H assay demonstrating SRAS1.1 interacts with SRAS1.2. Yeast transformants were grown on the DDO media and on the QDO+X-α-gal, greenish blue indicates positive interactions. (B) BiFC assay of interaction of SRAS1.1 with SRAS1.2. Yellow fluorescence indicates positive interactions. cYFP and nYFP was used as a negative control, (Scale bar, 20 μm).
(TIF)

S6 Fig The expression levels of CSN5A under salt stress. (A) qRT-PCR analysis of the expression levels of *CSN5A* in WT under 200mM NaCl treatments. The data were normalized to *GAPDH* and *UBQ10*. The means were calculated from three independent replicates and compared with the no-treatment condition (0 h). (B) qRT-PCR analysis of the expression levels of *CSN5A* in WT, *35S*::*SRAS1*.*1*, *35S*::*SRAS1*.*2* and *sras1-1*. Data are represented as means ± SD. The experiments were repeated at least three times with similar results. (C) Quantitative analysis of the signal intensity in Fig 7B. The abundance of CSN5A at the 0 hour was set to 1 as a reference for calculating relative abundance of various time point. Error bars indicate SEM (n = 3).
(TIF)

S7 Fig CSN5B does not interact with SRAS1.1 in plant. (A) Y2H assay demonstrating CSN5B interacts with SRAS1.1 and SRAS1.2. Yeast transformants were grown on the DDO media and on the QDO+X-α-gal, greenish blue indicates positive interactions. Proteins CSN6A and CSN6B were also tested. (B) Co-IP assay showing CSN5B cannot interact with SRAS1.1 *in vivo*. The construct combinations were expressed in *N*. *benthamiana* leaves. Total proteins were extracted and immunoprecipitated with anti-GFP agarose beads. The proteins were detected with anti-GFP and anti-CSN5 antibodies. (C) Cell-free assays showing the degradation rate of CSN5B-His incubated with the supernatant of WT, *sras1-1* and *35S*::*SRAS1*.*1#14*. The degradation rate of CSN5A-His was detected by anti-His antibody. Ponceau staining of Rubisco indicates equal loading. (D) Normalized plot of CSN5B-His contents based on the band intensities shown in (C). Error bars indicate SEM (n = 3).
(TIF)

S8 Fig The protein levels of SRAS1.1 and SRAS1.2 under salt stress. Immunoblot analysis of SRAS1 protein levels in 35S::GFP, 35S::SRAS1.1-GFP and 35S::SRAS1.2-GFP seedlings. The seedlings grew on 1/2 MS for 7 days. Then seedlings were treated with or without 200 mM NaCl for 6 h. Total proteins were extracted from different seedlings. The anti-GFP was used to detect GFP proteins. ACTIN served as a loading control. (B) Quantitative analysis of the signal intensity in (A). The abundance of GFP at the 0 mM NaCl treatment was set to 1 as a reference for calculating relative abundance of various time points. Error bars indicate SEM (n = 3).
(TIF)

S1 Table Differentially expressed genes in RNA-seq analysis. (XLSX)

S2 Table The detailed information of *SRAS* genes. (XLSX)

S3 Table Primers used in this study. (DOCX)

S4 Table *SRAS1.1* and *SRAS1.2* CDS. (DOCX)
