# Supplementary figures and images for: Salt responsive alternative splicing of a RING finger E3 ligase modulates the salt stress tolerance by fine-tuning the balance of COP9 signalosome subunit 5A
Source: PLoS Genet. 2021 Nov 16;17(11):e1009898. doi: 10.1371/journal.pgen.1009898 (PMC8631661; doi:10.1371/journal.pgen.1009898)

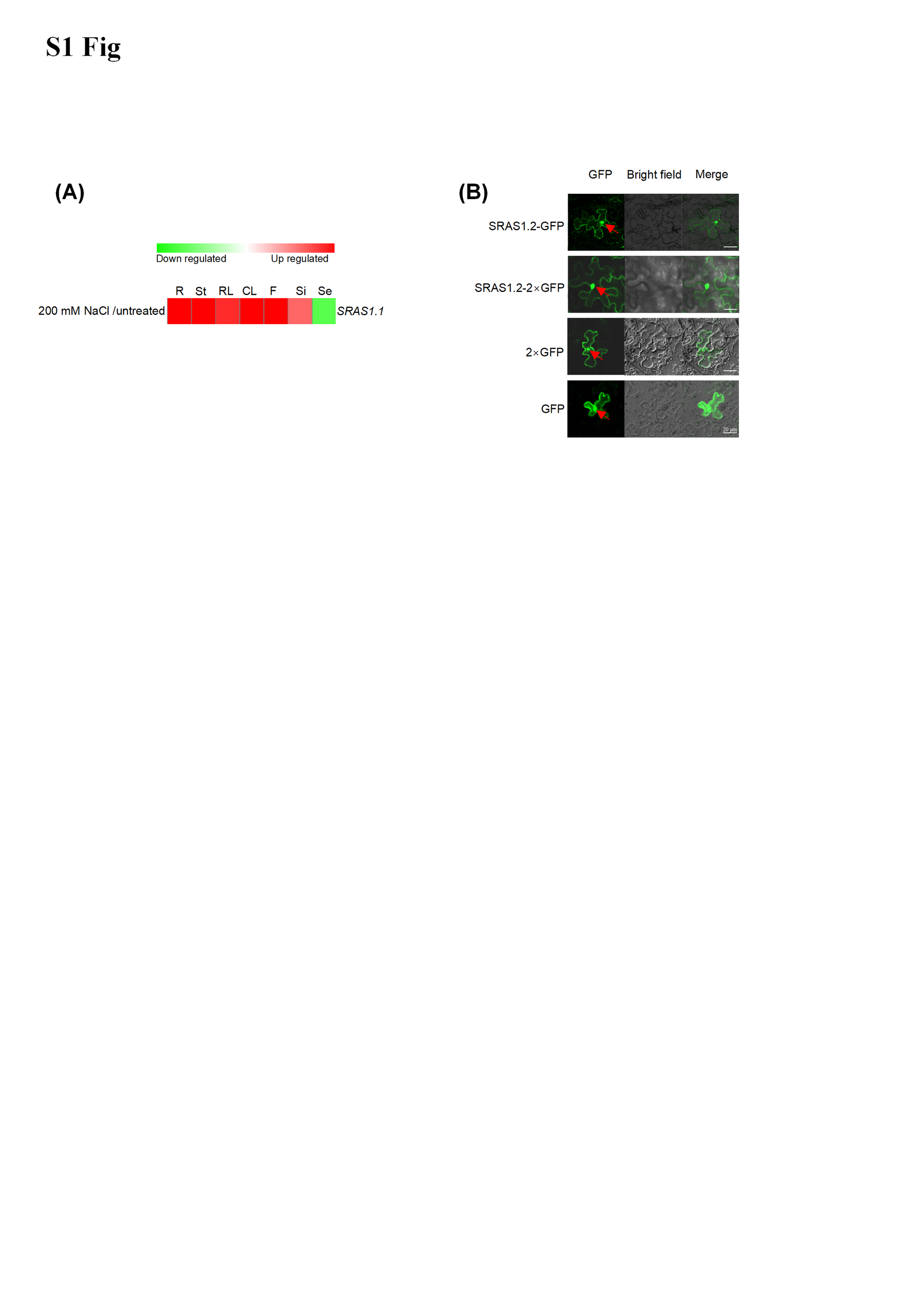

Supplement: S1 Fig — (A) Expression analysis of SRAS1 genes in various tissues under salt stress in Arabidopsis. Expression analysis was carried out with mRNA-seq datasets using Genevestigator. R: root, St: stem, RL: rosette leaf, CL: cauline leaf, F: flower, Si: silique, Se:seed. (B) Subcellular localization of SRAS1.2. The SRAS1.2-GFP, SRAS1.2–2×GFP plasmids were transformed into tobacco leaf cells. The fluorescence signals were collected from tobacco epidermal cells. GFP fluorescence (left) is shown. Bars = 20μm. (TIF) [file pgen.1009898.s001.tif]

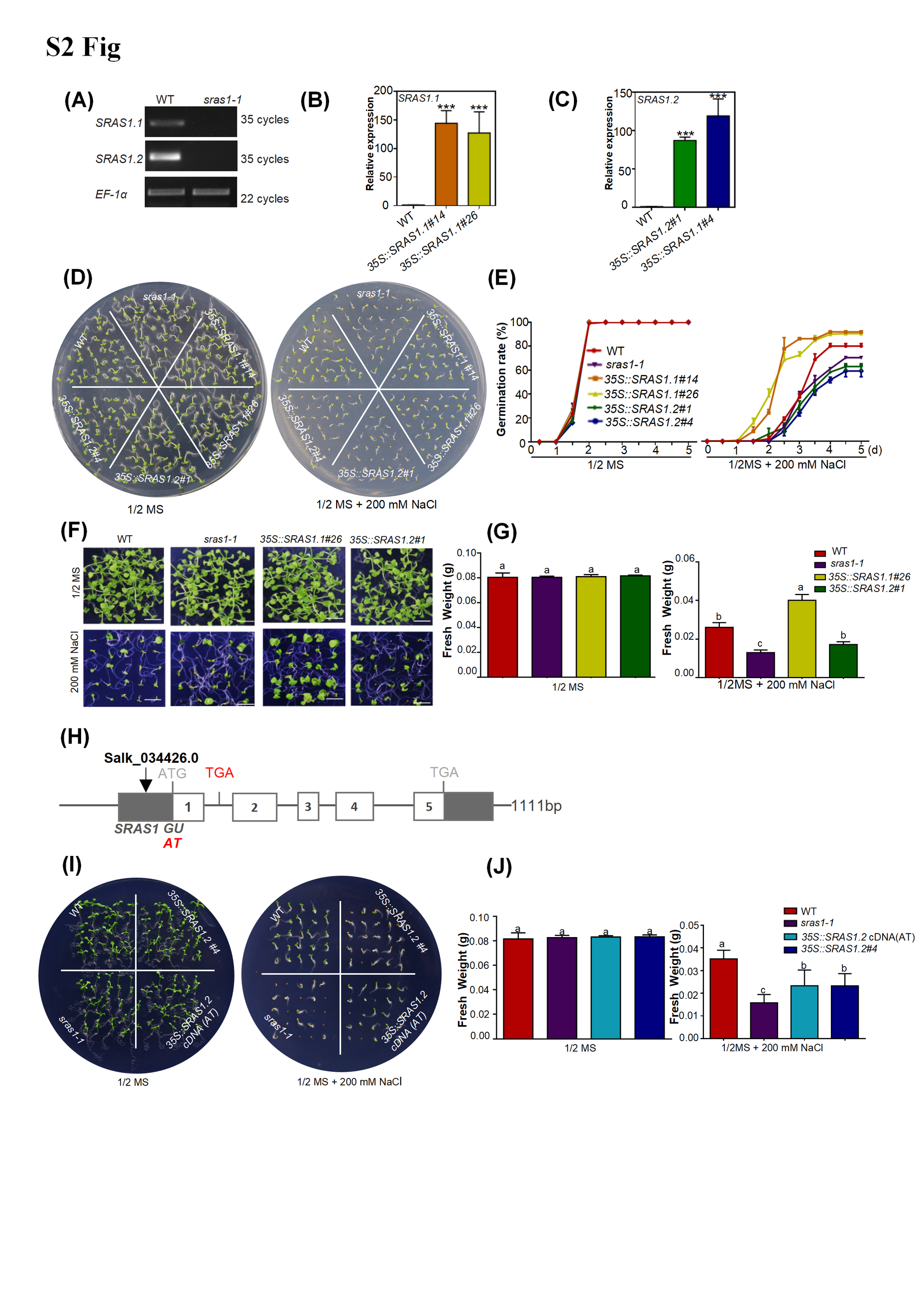

Supplement: S2 Fig — (A) RT-PCR analysis of the SRAS1.1 and SRAS1.2 transcripts in WT, sras1-1 mutant plants. EF-1α was used as a loading control. (B) qRT-PCR analysis of the expression of SRAS1.1 in WT, 35S::SRAS1.1#14 and 35S::SRAS1.1#26 seedlings. UBQ10 was used as a reference gene. Values are mean ± SE from three biological repeats. (C) qRT-PCR analysis of the expression of SRAS1.2 in WT, 35S::SRAS1.2#1 and 35S::SRAS1.2#4 seedlings. UBQ10 was used as a reference gene. Values are mean ± SE from three biological repeats. (D) Germination phenotype of WT, 35S::SRAS1.1#14, 35S::SRAS1.1#26, 35S::SRAS1.2#1, 35S::SRAS1.2#4 and sras1-1 seedlings grown on 1/2 MS medium with or without 200 mM NaCl. Images were taken 7 days after germination. (E) The germination rates of WT, 35S::SRAS1.1#14, 35S::SRAS1.1#26, 35S::SRAS1.2#1, 35S::SRAS1.2#4 and sras1-1 seedlings grown on 1/2 MS medium with or without 200 mM NaCl. The values are the mean ± standard deviation from three biological replicates. (F) Fresh weight of 35S::SRAS1.1, 35S::SRAS1.2, WT and sras1-1 seedlings growing 1/2 MS agar plates with or without 200 mM NaCl photographs were taken after growing at 22°C for 14 d. Bar = 1 cm. (G) The quantification of relative fresh weight of 35S::SRAS1.1, 35S::SRAS1.2, WT and sras1-1 seedlings growing 1/2 MS agar plates with or without 200 mM NaCl. The bars indicate means ± SD of three independent measurements. Different letters indicate that values were significantly different at P < 0.01. (H) Schematic illustration of the T-DNA insertion sites in the sras1-1 mutants. Gray boxes represent untranslated regions (UTRs), White boxes represent exons; line segments represent introns; black arrows represent insertion sites of the sras1-1 mutant alleles. GU or AT represent special mutation points in overexpressing SRAS1.2 transcript. (I) Germination phenotype of WT, sras1-1 35S::SRAS1.2#4 and 35S::SRAS1.2 cDNA(AT) seedlings grown on 1/2 MS medium with or without 200 mM NaCl. Images were taken 7 days after germina [file pgen.1009898.s002.tif]

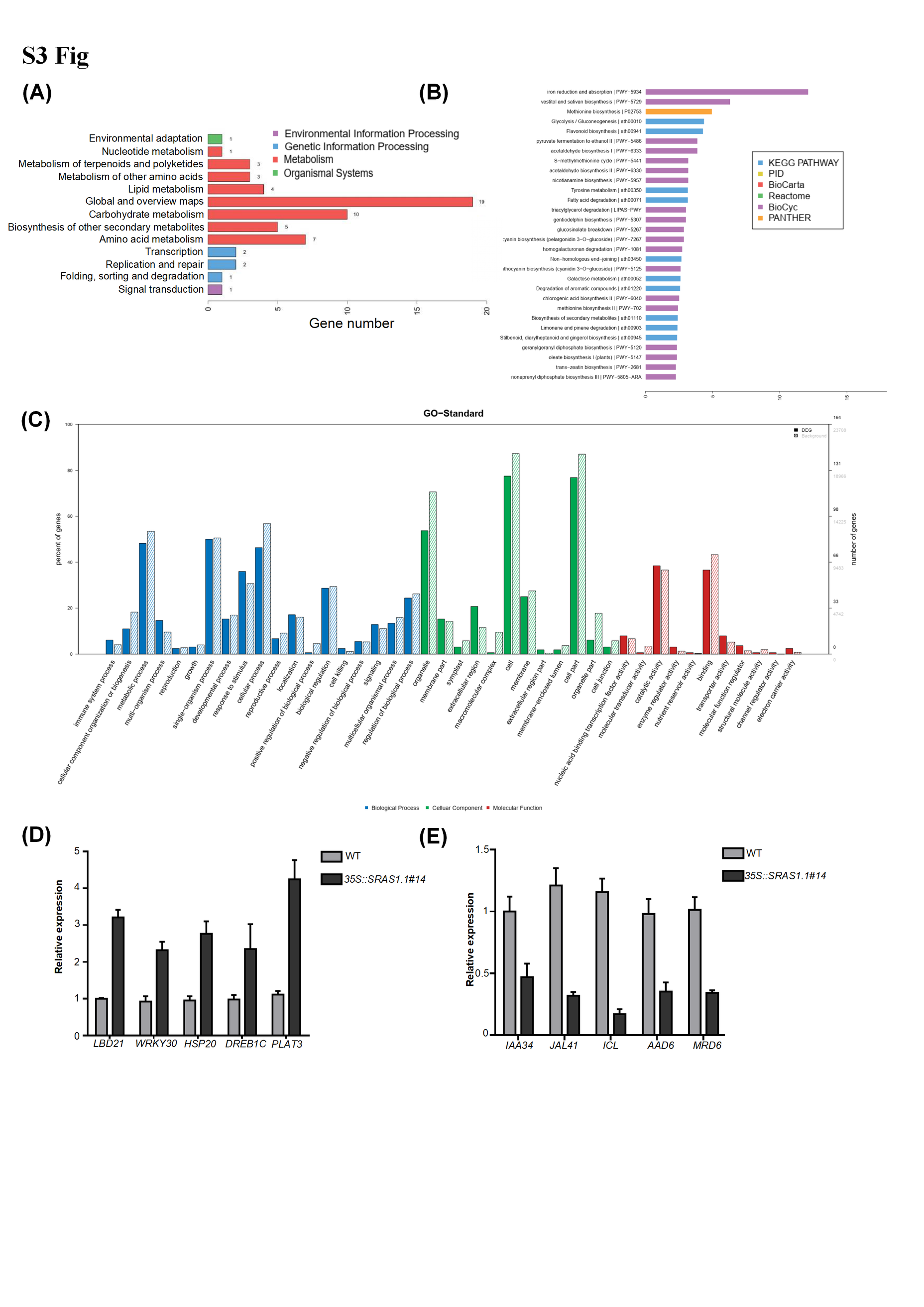

Supplement: S3 Fig — (A) Enrichment of differential genes in KEGG classification. (B) GO function significant enriched pathway terms. The abscissa is the negative log value of p-value, the ordinate is the first 30 enriched GO terms and function descriptions. (C) Gene ontology second-level entry frequency chart. The abscissa is the GO database function description, the ordinate is the number of genes. Different biological functions are shown in red, blue and green bars, respectively. (D)-(E) qRT-PCR analysis of the expression levels of marker genes involved salt response pathways. Data are represented as means ± SD, n = 6. **P < 0.01**P < 0.001. (TIF) [file pgen.1009898.s003.tif]

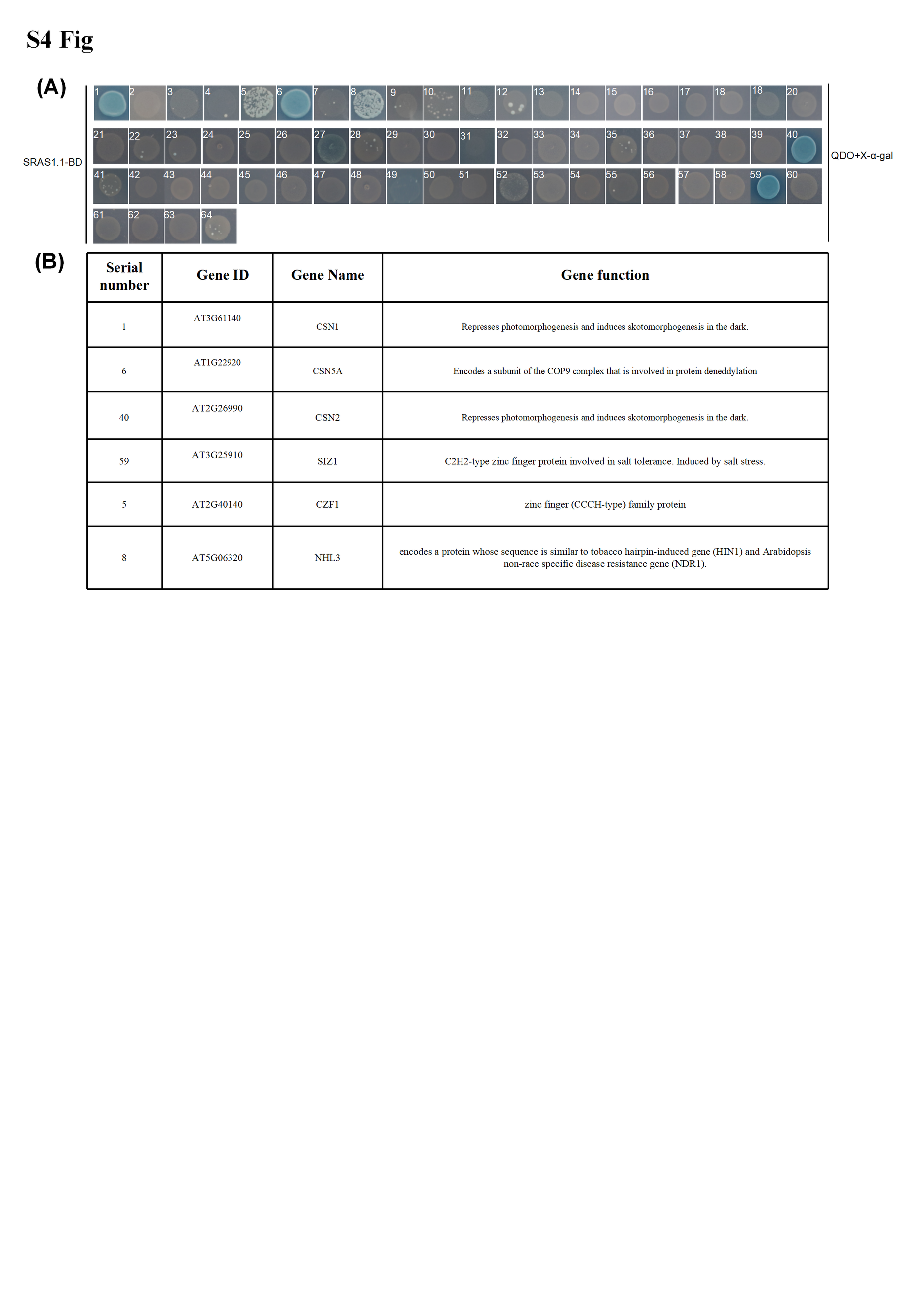

Supplement: S4 Fig — Interactions between SRAS1.1 and target proteins in Y2H assays. Yeast transformants were grown on the DDO media and on the QDO+X-α-gal, greenish blue indicates positive interactions, greenish blue indicates positive interactions. (B) Detailed information of SRAS1.1 interaction proteins. The SRAS1.1-interacting proteins, plasmids were recovered from yeast strains showing positive interactions, and their sequences were verified by DNA sequencing. Sequence data for the proteins described found in the Arabidopsis TAIR database. (TIF) [file pgen.1009898.s004.tif]

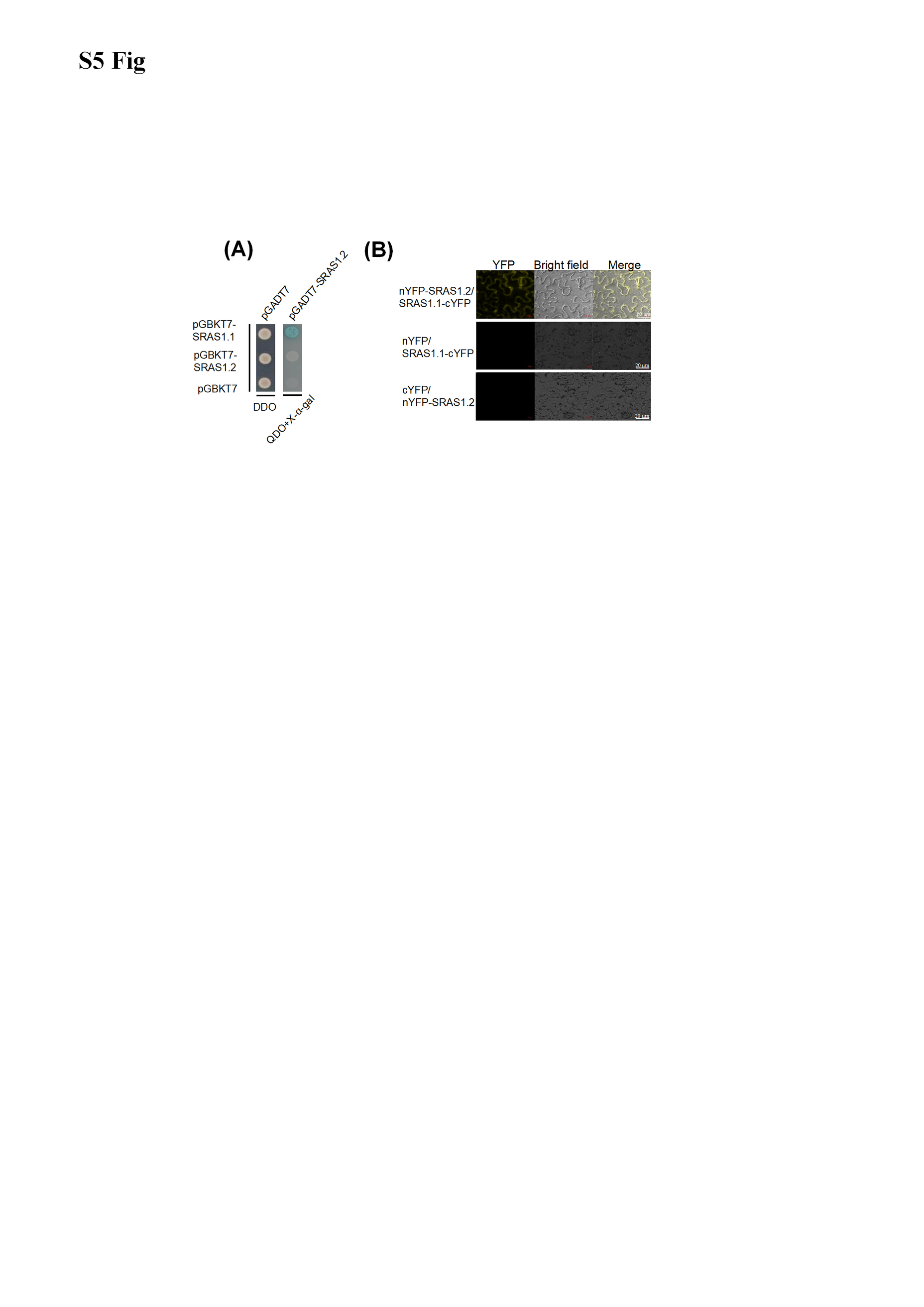

Supplement: S5 Fig — (A) Y2H assay demonstrating SRAS1.1 interacts with SRAS1.2. Yeast transformants were grown on the DDO media and on the QDO+X-α-gal, greenish blue indicates positive interactions. (B) BiFC assay of interaction of SRAS1.1 with SRAS1.2. Yellow fluorescence indicates positive interactions. cYFP and nYFP was used as a negative control, (Scale bar, 20 μm). (TIF) [file pgen.1009898.s005.tif]

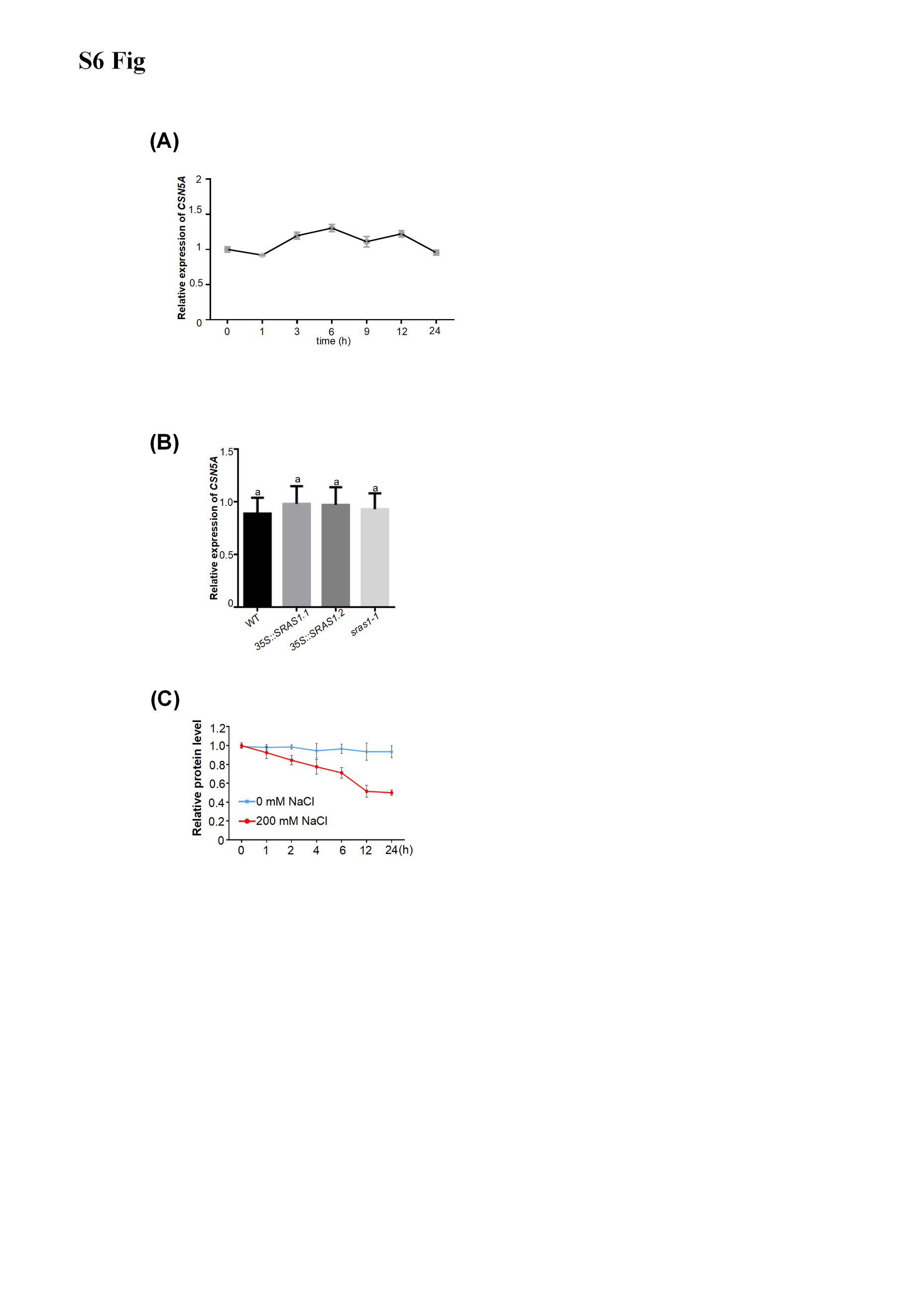

Supplement: S6 Fig — (A) qRT-PCR analysis of the expression levels of CSN5A in WT under 200mM NaCl treatments. The data were normalized to GAPDH and UBQ10. The means were calculated from three independent replicates and compared with the no-treatment condition (0 h). (B) qRT-PCR analysis of the expression levels of CSN5A in WT, 35S::SRAS1.1, 35S::SRAS1.2 and sras1-1. Data are represented as means ± SD. The experiments were repeated at least three times with similar results. (C) Quantitative analysis of the signal intensity in Fig 7B. The abundance of CSN5A at the 0 hour was set to 1 as a reference for calculating relative abundance of various time point. Error bars indicate SEM (n = 3). (TIF) [file pgen.1009898.s006.tif]

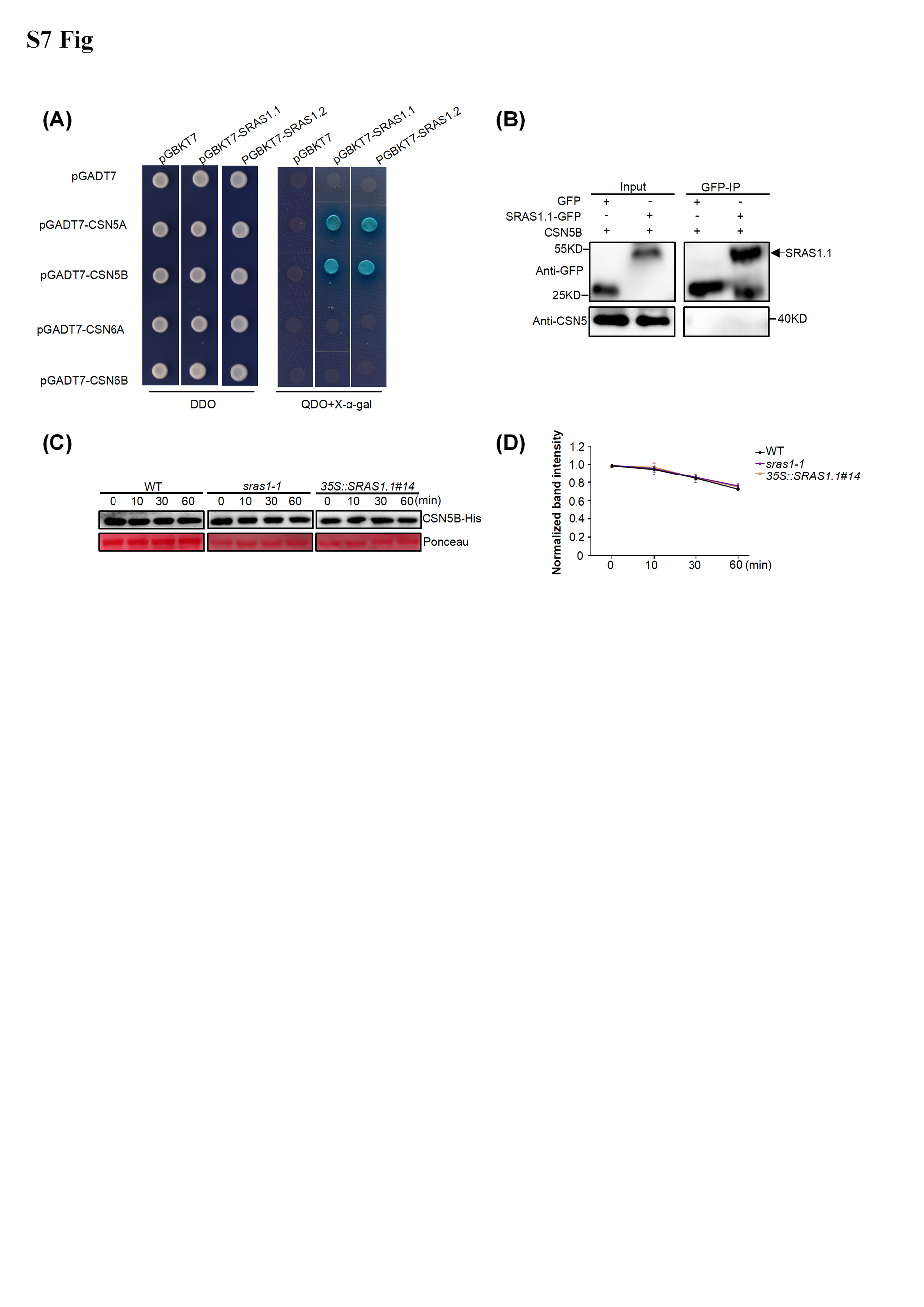

Supplement: S7 Fig — (A) Y2H assay demonstrating CSN5B interacts with SRAS1.1 and SRAS1.2. Yeast transformants were grown on the DDO media and on the QDO+X-α-gal, greenish blue indicates positive interactions. Proteins CSN6A and CSN6B were also tested. (B) Co-IP assay showing CSN5B cannot interact with SRAS1.1 in vivo. The construct combinations were expressed in N. benthamiana leaves. Total proteins were extracted and immunoprecipitated with anti-GFP agarose beads. The proteins were detected with anti-GFP and anti-CSN5 antibodies. (C) Cell-free assays showing the degradation rate of CSN5B-His incubated with the supernatant of WT, sras1-1 and 35S::SRAS1.1#14. The degradation rate of CSN5A-His was detected by anti-His antibody. Ponceau staining of Rubisco indicates equal loading. (D) Normalized plot of CSN5B-His contents based on the band intensities shown in (C). Error bars indicate SEM (n = 3). (TIF) [file pgen.1009898.s007.tif]

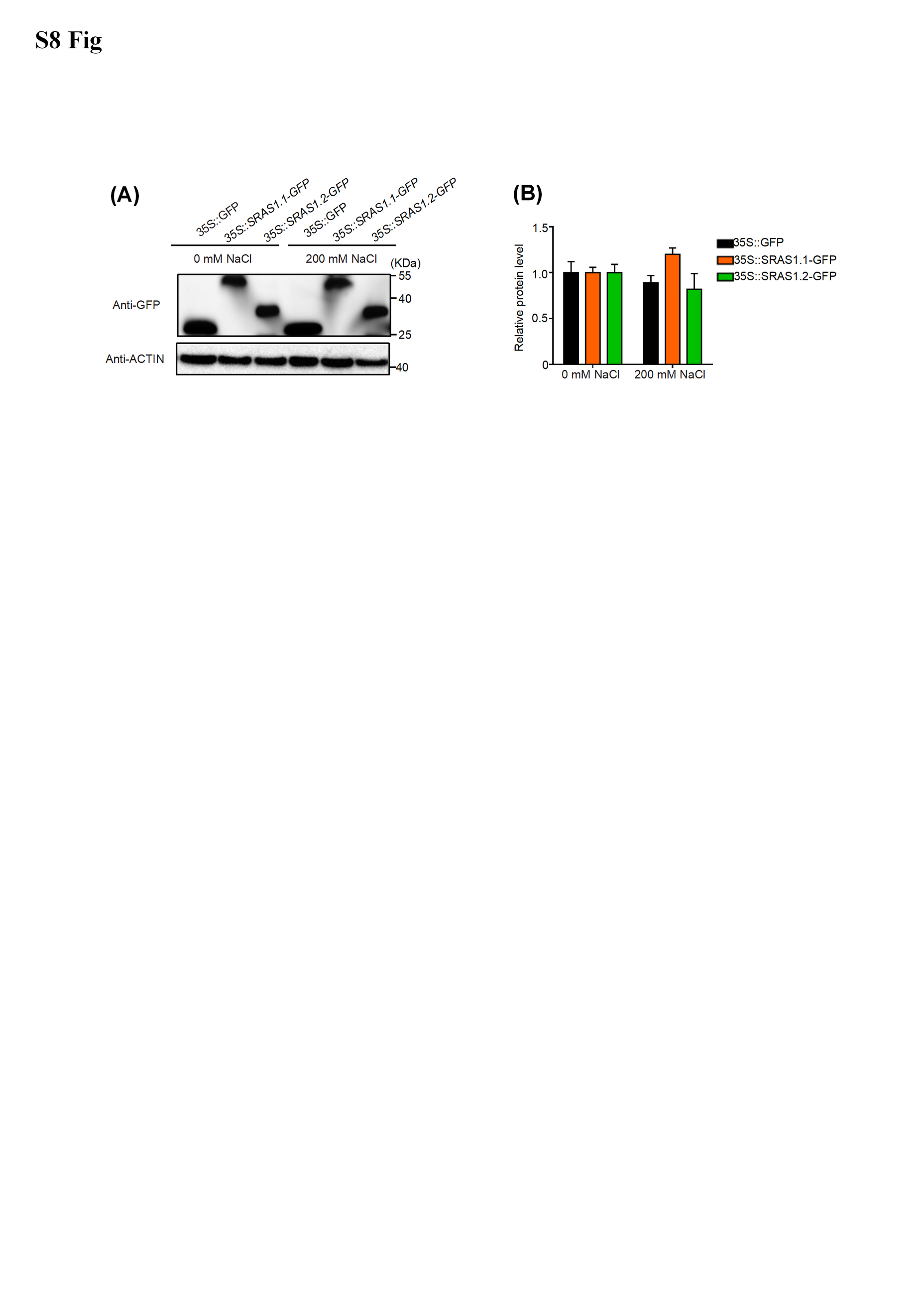

Supplement: S8 Fig — Immunoblot analysis of SRAS1 protein levels in 35S::GFP, 35S::SRAS1.1-GFP and 35S::SRAS1.2-GFP seedlings. The seedlings grew on 1/2 MS for 7 days. Then seedlings were treated with or without 200 mM NaCl for 6 h. Total proteins were extracted from different seedlings. The anti-GFP was used to detect GFP proteins. ACTIN served as a loading control. (B) Quantitative analysis of the signal intensity in (A). The abundance of GFP at the 0 mM NaCl treatment was set to 1 as a reference for calculating relative abundance of various time points. Error bars indicate SEM (n = 3). (TIF) [file pgen.1009898.s008.tif]
