# Supplementary material for: Salt responsive alternative splicing of a RING finger E3 ligase modulates the salt stress tolerance by fine-tuning the balance of COP9 signalosome subunit 5A
Source: PLoS Genet. 2021 Nov 16;17(11):e1009898. doi: 10.1371/journal.pgen.1009898 (PMC8631661; doi:10.1371/journal.pgen.1009898)
Supplement: S3 Table — (DOCX) [file pgen.1009898.s011.docx]

| Primer name | Sequence (5' to 3' ) | Applycation |
| --- | --- | --- |
| F1 | F:5′-ATGGATGGTTATTATTCTCTGTCTCCC-3’ | Identification of *SRAS1* |
| R1 | R:5′-TGAAGGCAGACAGAGCAAGAGAC -3′ |  |
| F2 | F:5-TTCTTCGCTTTAGTGGGGACTTTGC-3’ |  |
| R2 | R:5′-CTCAAAAGGCTAGCAATGACATCAAT-3’ |  |
| F3 | F:5-TCCTTGTTTCTGCAATTACGATATGAA-3’ |  |
| R3 | R:5′-CTCAAAAGGCTAGCAATGACATCAAT-3’ |  |
| SRAS1.1-BD | F:5′-GAATTCATGGATGGTTATTATTCTCTGTCTCCCAT-3’ | Yeast two-hybrid |
|  | R:5′-CTCGAGAAGATGTCTTCTGCACAAGGGACA -3′ |  |
| SRAS1.2-BD | F:5′-GAATTCATGGATGGTTATTATTCTCTGTCTCCCAT-3’ |  |
|  | R:5′-CTCGAGGGGATCACAGAGATTGATTAATGAAGATG-3′ |  |
| SRAS1.1-AD | F:5′-GAATTCATGGATGGTTATTATTCTCTGTCTCCCAT -3′ |  |
|  | R:5′-CTCGAGAAGATGTCTTCTGCACAAGGGACA-3′ |  |
| SRAS1.2-AD | F:5′-GAATTCATGGATGGTTATTATTCTCTGTCTCCCAT-3′ |  |
|  | R:5′-CTCGAGGGGATCACAGAGATTGATTAATGAAGATG-3′ |  |
| CSN5A-AD | F:5′-GAATTCATGGAAGGTTCCTCGTCAGCC-3′ |  |
|  | R:5′-GGATCCCACGATGTAATCATGGGCTCTGGAT-3′ |  |
| CSN5B-AD | F:5′- GAATTCATGGAGGGTTCGTCGTCGA-3′ |  |
|  | R:5′-GGATTCATATGTAATCATAGGGTCTGGATCCG-3′  -3′ |  |
| 35S:*SRAS1.1* | F:5′-GAATTCATGGATGGTTATTATTCTCTGTCTCCCAT-3’ | Vector construction |
|  | R:5′-CTCGAGAAGATGTCTTCTGCACAAGGGACA -3′ |  |
| 35S:*SRAS1.2* | F:5′-GGTACCATGGGGACAGGGAATTCTAAAGAAAAC-3′ |  |
|  | R:5′-CTCGAGAAGATGTCTTCTGCACAAGGGACA -3′ |  |
| 35S:*SRAS1.2* | F:5′-GGTACCATGGGGACAGGGAATTCTAAAGAAAAC-3′ |  |
|  | R:5′-CTCGAGTCAGGGATCACAGAGATTGATTAAT -3′ |  |
| *sras1-1* | F:5′-CTGACTCTGGACGGCACTTAG-3′ | T-DNA insertion |
|  | R:5′-GTACAGGGAAAGGTGAGGAGG-3′ |  |
| *csn5a-2* | F:5′- ACGATGTAATCATGGGCTCTG-3′ |  |
|  | R:5′-TCACCTTCTGGATCTCCTTTG-3′ |  |
| LBb1.3 | F:5′-ATTTTGCCGATTTCGGAAC-3′ |  |
| GST-SRAS1.1 | F:5′-GAATTCCATGGATGGTTATTATTCTCTGTCTCCCAT-3′ | Pull-dwon |
|  | R:5′-CTCGAGTCGAAGATGTCTTCTGCACAAGGGACA-3′ |  |
| GST-SRAS1.2 | F:5′-GAATTCCATGGATGGTTATTATTCTCTGTCTCCCAT-3′ |  |
|  | R:5′-CTCGAGGGGATCACAGAGATTGATTAATGAA-3′ |  |
| CSN5A-His | F:5′-GGATCCCGGGATCCATGGAAGGTTCCTCGTCAGCC-3′ |  |
|  | R:5′-GTCGACCGATGTAATCATGGGCTCTGGATCT-3′ |  |
| CSN5B-His | F:5′-GAATTCATGGAGGGTTCGTCGTCGA-3′  -3′ |  |
|  | R:5′-AAGCTTATATGTAATCATAGGGTCTGGATCCG-3′ |  |
| CSN5B-GST | F:5′-GAATTCATGGAGGGTTCGTCGTCGA-3′ |  |
|  | R:5′-CTCGAGATATGTAATCATAGGGTCTGGATCCG-3′ |  |
| SRAS1.1-cYFP | F:5′-TCTAGAATGGATGGTTATTATTCTCTGTCTCCCATCT-3′ | BiFC |
|  | R:5′-GGTACCAAGATGTCTTCTGCACAAGGGACAA-3′ |  |
| SRAS1.2-cYFP | F:5′-TCTAGAATGGATGGTTATTATTCTCTGTCTCCCATCT-3′ |  |
|  | R:5′-GGTACCGGGATCACAGAGATTGATTAATGAA-3′ |  |
| nYFP-SRAS1.2 | F:5′-GAATTCATGGTTATTATTCTCTGTCTCCCATCT-3′ |  |
|  | R:5′-GTCGACTCACAGAGATTGATTAATGAA-3′ |  |
| nYFP-CSN5A | F:5′-GAATTCATGGAAGGTTCCTCGTCAGCC-3′ |  |
|  | R:5′-GTCGACCGATGTAATCATGGGCTCTGGATCT-3′ |  |
| SRAS1.1-cLUC | F:5′-GGTACCATGGATGGTTATTATTCTCTGTCTCCCATCTCTG-3′ | LCI |
|  | R:5′-GTCGACTCAAAGATGTCTTCTGCACAAGGGACAAGA-3′ |  |
| SRAS1.2-cLUC | F:5′-GGTACCATGGATGGTTATTATTCTCTGTCTCCCATCTCTG-3′ |  |
|  | R:5-GTCGACTCAGGGATCACAGAGATTGATTAGAAGATGAA-3′ |  |
| nLUC-CSN5A | F:5′-GGTACCATGGAAGGTTCCTCGTCAGCC-3′ |  |
|  | R:5-GTCGACCGATGTAATCATGGGCTCTGG-3′ |  |
| SRAS1.1-GFP | F:5′-AAGCTTATGGATGGTTATTATTCTCTGTCTCCC-3′ | Subcellular  localization |
|  | F:5′-GGTACCAAGATGTCTTCTGCACAAGGGAC-3 |  |
| SRAS1.2-GFP | F:5′-AAGCTTATGGATGGTTATTATTCTCTGTCTCCC-3′ |  |
|  | F:5′-GGTACCGGGATCACAGAGATTGATTAATGAA-3′ |  |
| SRAS1^RING^-GFP | F:5′-AAGCTTTCTCTTGCTCTGTCTGCCTTCAG-3′ |  |
|  | F:5′-GGTACCAAGATGTCTTCTGCACAAGGGACA-3′ |  |
| SRAS1.2-GFP dimer | F:5′-AAGCTTATGGATGGTTATTATTCTCTGTCT-3′ |  |
|  | F:5′-GGTACCCTTGTACAGCTCGTCCATGCC-3′ |  |
| *SRAS1.1* | F:5-TTCTTCGCTTTAGTGGGGACTTTGC-3’ | qRT and RT |
|  | R:5′-CTCAAAAGGCTAGCAATGACATCAAT-3’ |  |
| *SRAS1.2* | F:5′-GTTCCTTCTTTTCTTCACATTCATCT-3’ |  |
|  | R:5′-CTCAAAAGGCTAGCAATGACATCAAT-3’ |  |
| *CSN5A* | F:5′-ATGGAAGGTTCCTCGTCAGCC-3′ |  |
|  | R:5′-CGATGTAATCATGGGCTCTGGATCT-3′ |  |
| *ABI5* | F:5′-ATGGTAACTA GAGAAACGAAGTT-3′ |  |
|  | R:5′-CTCGGGTCCTCATCAATGTCCGCAA-3′ |  |
| *WRKY30* | F:5′-CGAGCTAATG ATAGAAGGAA-3′  151 GAGACT-3′ |  |
|  | R:5′-CAAATGTGCTTGGTGATTGTAGT-3′ |  |
| *DREB1C* | F:5′-CTCAT TTTCTGCCTT TTCTGAA-3′  151 GAGACT-3′ |  |
|  | R:5′-TCGTCATATGACACATCTCATCTTGA-3′ |  |
| *IAA34* | F:5′-ATCCCTTGCACTTAGTGGCATCAGA-3′  151 GAGACT-3′ |  |
|  | R:5′-CACTTCCGTCTGTATCCTTCATTAC-3′ |  |
| *ICL* | F:5′-AGGCTATGCTTCGAACGAGATGGCTA-3′  151 GAGACT-3′ |  |
|  | R:5′-ATGTTCGCTGACTGCCACGAGGACCTTT-3′ |  |
| *LBD21* | F:5′-ATGAGAGGGCATGAGCCACG-3′  151 GAGACT-3′ | qRT and RT |
|  | R:5′-CTATACAAAAGGAGGCTGTCCA3′ |  |
| *PLAT3* | F:5′-ATGAGCCTCCGTCTTTACGACAG-3′  151 GAGACT-3′ |  |
|  | R:5′-CTACAGCTCATACGGTGAAGCATCA3′ |  |
