# Supplementary material for: Salt responsive alternative splicing of a RING finger E3 ligase modulates the salt stress tolerance by fine-tuning the balance of COP9 signalosome subunit 5A
Source: PLoS Genet. 2021 Nov 16;17(11):e1009898. doi: 10.1371/journal.pgen.1009898 (PMC8631661; doi:10.1371/journal.pgen.1009898)
Supplement: S4 Table — (DOCX) [file pgen.1009898.s012.docx]

**SRAS1.1 CDS 666 bp**

**(a)**

1 ATGGATGGTT ATTATTCTCT GTCTCCCATC TCTGTCCTCC ACCGGATTAA AGATTCCTTC CATTTCGCCG TCTCTGCCCT TCTCGCCAAC CTCTTCTCCG

101 CTCTCTTCAC CTTCTTCTTC GCTTTAGTGG GGACTTTGCT GGGAGCATTG ACAGGGGCTT TGATCGGCCA AGAAACAGAG AGCGGTTTCA TCAGAGGAGC

201 CGCCGTTGGT GCTATCTCAG GCGCCGTCTT CTCCATCGAA GTCTTTGAAT CTTCCCTCCT CCTTTGGCAA TCCGATGAGT CTGGAATTGG ATGCCTTCTC

301 TACTTGATTG ATGTCATTGC TAGCCTTTTG AGCGGGAGGC TTGTTCGTGA GCGTATCGGT CCTGCAATGC TAAGTGCCGT CCAGAGTCAG ATGGGAGCTG

401 TGGAGTCCCA GTTCCAAGAT CATACAGACA TCTTTGACAC TGCCATTTCA AAGGGTCTCA CTGGGGACTC TCTCAACAGG ATCCCTAAGG TCCGAATCAC

501 AGACACCTCT CCGGAGATTG TCTCTTGCTC TGTCTGCCTT CAGGACTTTC AGGTGGGAGA GACAGTTAGA AGTTTGCCGC ACTGCCATCA TATGTTCCAC

601 CTACCATGCA TCGACAAATG GCTTCGCAGG CATGCTTCTT GTCCCTTGTG CAGAAGACAT CTTTGA

**(b)**

**SRAS1.2 CDS 180 bp**

1. 1 ATGGATGGTT ATTATTCTCT GTCTCCCATC TCTGTCCTCC ACCGGATTAA AGATTCCTTC CATTTCGCCG TCTCTGCCCT TCTCGCCAAC CTCTTCTCCG

101 CTCTCTTCAC CTTCTTCTTC GCTTTAGGTT CCTTCTTTTC TTCACATTCA TCTTCATTAA TCAATCTCTG TGATCCCTGA

**SRAS1.2 cDNA 1305 bp**

**(c)**

**Point mutations at the 5' splice site (from GU to AT) in the second intron**

1 GATTAAATTG GAAGCCAAAA AAGTCAGAAG AAGGGCGCAA AAGTCTTACC CACTCATCGT TTTCTTTAAA TAATCAAATC CACTCGTCGT CGTAAGATAT
101 TTTCCTTTCG AATATTAAGA AAGATGAGAC GCGTGATTAT TAATTAATAA AGAAACTTTC GGTCAGCGGA GAGAAGGTTC CTTTGCCTTT GGTCTCTTTT
201TCTAATCGAT TGATTATATC TTCAAATAAA AAGGAGAAAA GAAAGGTTCC TTGTTTTTCC AGACAAAGAA AACACAAATT CATTCCCTCC TCCTCTTCAT
301 CTCTTTTGCA GATATGGATG GTTATTATTC TCTGTCTCCC ATCTCTGTCC TCCACCGGAT TAAAGATTCC TTCCATTTCG CCGTCTCTGC CCTTCTCGCC
401 AACCTCTTCT CCGCTCTCTT CACCTTCTTC TTCGCTTTAG GTTCCTTCTT TTCTTCACAT TCATCTTCAT TAATCAATCT CTGTGATCCCT GAGGAATTT

501 TCCTTGTTTCTGCAATTACGATATGAATATGATTTGACTTCTTTGTTGATTTGATTCAGTGGGGACTTTGCTGGGAGCA TTGACAGGGG CTTTGATCGGC

601 CAAGAAACAGAGAGCGGTTTCATCAGAGGAGCCGCCGTTGGTGCTATCTCAGGCGCCGTCTTCTCCATCGAAGTCTTTG AATCTTCCCT CCTCCTTTGGC

701 AATCCGATG AGTCTGGAATTGGATGCCTT CTCTACTTGA TTGATGTCAT TGCTAGCCTT TTGAGCGGGAGGCTTGTTCG TGAGCGTATC GGTCCTGCAA T

801 GCTAAGTGCCGTCCAGAGTCAGATGGGAGCTGTGGAGTCCCAGTTCCAAGATCATACAGACATCTTTGACACTGCCATT TCAAAGGGTC TCACTGGGGAC

901 TCTCTCAACAGGATCCCTAAGGTCCGAATCACAGACACCTCTCCGGAGATTGTCTCTTGCTCTGTCTGCCTTCAGGACT TTCAGGTGGG AGAGACAGTT A

1001 GAAGTTTGCCGCACTGCCATCATATGTTCCACCTACCATGCATCGACAAATGGCTTCGCAGGCATGCTTCTTGTCCCTT GTGCAGAAGA CATCTTTGAA T

1101 TGATTTTGAACTCTTCTTTCTTCTCTTCTTTGTACATGAGGTATAGGCATACACACACACACATATATACACACTAGTA GTCTTCCTTG ATTTTTTTTAG

1201 ATTACACGT TACACAGATTTTGATGCACC AATCATTGTA ACTTCACTCA TCTCTTGGAG ATCCTGTTTA TTTATGCTAC TTATTCTTGG CTTCATCATT C

1301 AAAAA

AT
